# Neuronal Microtubules and Radiofrequency Waves: The Quantum Core of Human Consciousness, Memory, and Pathway to Memory Enhancement/Recovery

**DOI:** 10.3390/ijms27136090

**Published:** 2026-07-07

**Authors:** Gary W. Arendash

**Affiliations:** 1 Department of Molecular Biosciences, University of South Florida, Tampa, FL 33620, USA; arendash@usf.edu; Tel.: +1-(480)-395-1481; 2 RF Longevity SE, 428 E. Thunderbird Rd., Phoenix, AZ 85022, USA

**Keywords:** microtubules, radiofrequency waves, superposition qubits, biophotons, consciousness, memory, Alzheimer’s disease, orchestrated objective reduction, Environmentally-Induced Decoherence

## Abstract

A unifying theory of both human consciousness and memory is presented that is based on neuronal microtubules (MTs) being central to both, and different populations of pyramidal cells in neocortex and hippocampus being responsible for consciousness or memory. First, two quantum theories of consciousness are presented—the Orchestrated Objective Reduction (Orch OR) theory of Penrose/Hameroff and the Environmental-Induced Decoherence Theory (EID) theory of Neven. A Hybrid (MT/EID) theory is proposed in the context of “consciousness”-dedicated pyramidal cells in Layer V of the cerebral cortex. This MT/EID theory involves the MT vibrations of the Orch OR Theory, along with the continual superposition qubit (SPQ) formation and SPQ entanglement of the EID Theory to collectively induce SPQ formation/entanglement in Layer V of the cerebral cortex. Orch OR’s objective reduction (collapse of the waveform) is not included in this Hybrid theory because the system of SPQs themselves continuously collapses due to EID. For memory, it is proposed that Orch OR forms its basis with three specific modifications: (1) presenting “endogenous” radiofrequency (RF) vibrations generated by neuronal microtubules as forming a microtubule/RF wave “vibrational fabric” involving microtubular crystalline water cores, (2) refining Orch OR for memory by proposing SPQ formation for short-term memory and objective reduction of those qubits primarily in “memory-dedicated” pyramidal cells within cortical Layers II/III for long-term memory storage through “Quantum Darwinism” (SPQ/OR), and (3) integrating SPQ/OR with the ability of “externally” applied RF waves at 1 GHz to beneficially influence human memory through microtubule-enhancing mechanisms. It is proposed that a vibrational fabric consisting of MTs, RF waves, and generated photons provides the Photonic/RF-wave quantum coherence necessary for brain memory processing. Strong evidence for beneficial effects of exogenous RF wave treatment on memory is provided by a new bioengineered technology—Transcranial Radiofrequency Wave Treatment (TRFT; also known as TEMT). This evidence is presented in both pre-clinical and clinical studies involving normal and Alzheimer’s Disease (AD) transgenic mice, and AD patients bearing memory loss. In support of MT involvement in memory, TRFT would appear to be an ideal non-pharmacologic technology to beneficially modulate the microtubule/RF wave vibrational fabric—an intraneuronal fabric that may be at the deep core of human memory, and thus the key to Alzheimer’s Disease memory rescue.

## 1. Introduction

The basis of human consciousness and memory has been the subject of numerous theories and hypotheses, with proposed mechanisms of consciousness and memory being separate from one another and usually with little or no empirical scientific evidence for them. Prominent theories of human consciousness include Integrated Information Theory (IIT), Global Workspace Theory (GWT), and Global Neuronal Workspace Theory (GNWT) [1,2]. The IIT proposes that consciousness is a fundamental property of systems (e.g., the brain) that can integrate information, advocating a deep connection between consciousness and the physical structure of the brain. GWT is a somewhat vague scaffold proposing that information is mostly processed unconsciously, but becomes conscious only when it enters a central, limited-capacity “global workspace” that then broadcasts it to the rest of the brain. As an extension of GWT, the GNWT postulates that, in the conscious state, a “network ignition” associated with recurrent processing maintains a neural representation, thus allowing the consequent information to be globally accessed. The GNWT emphasizes the role of long-range cortico-cortical loops, which are linked with feedforward and feedback connections. These widespread corticocortical connections are hypothesized to originate from cortical layers II/III and V/VI and permit communication between widely distributed cortical processors. None of these consciousness theories appears to provide details below the levels of classical brain areas and neuronal activity/networks; rather, they have largely been explored through EEG and brain imaging (e.g., fMRI, MEG) studies. In 2025, the IIT and GNWT theories were compared to one another directly in empirical testing (e.g., MRI). The conclusion reached was that neither theory is fully capable of accounting for consciousness [3].

There are also a number of electromagnetic-based theories of consciousness that all purport electromagnetic waves as the seat of human consciousness [4], possibly involving coherent brain electromagnetic oscillations or an interplay between neuronal activity and electromagnetic fields. One of these theories posits that consciousness arises from the brain interacting with electromagnetic waves in the high giga-hertz (GHz) to tetra-hertz (THz) range that are ubiquitous within the zero point field filling all space in the universe [5,6]. Though attractive in concept, this theory is untenable because electromagnetic waves at those high frequencies cannot penetrate the human skull to affect the brain. As such, it would seem unlikely that consciousness arises from the universe itself—this, with or without involvement of electromagnetic waves. Despite no experimental evidence for it, the current in-vogue popularity of consciousness surrounding us is being driven by a few theoretical physicists. As with other theories of consciousness, electromagnetic-based theories lack specifics and empirical evidence [4].

Similar to theories of consciousness, proposed theories of human memory largely involve neuronal networks and the connections (synapses) between their component neurons [7]. The primary “cellular/sub-cellular”-based theories of memory include: (1) The Synaptic Plasticity Theory, whereby memory encoding is thought to involve synaptic long-term potentiation (LTP) or long-term depression (LTD), followed by synaptic plasticity and neuronal structural changes for long-term storage [8], (2) The Engram Cell Theory, which postulates that memory is stored and retrieved through a subset of neurons called “engram cells” that are very active during a memory event and are thus allocated the event’s memory trace, which stores event information in a stable state that is then reactivated during recall of the event [9], (3) The Epigenetic Coding Theory, which posits that memory is stored by modifying the DNA within neurons to create a permanent molecular record (epigenetic marks) of a past event that can be stored [10], and (4) The Intrinsic Plasticity Theory, which purports that neurons can modify their level of excitability, making them more or less likely to be activated by future inputs [11]. All of these theories would presumably involve the major brain areas for memory encoding, consolidation, and storage/retrieval—the hippocampus (and adjacent entorhinal cortex, amygdala), prefrontal cortex, and the vast neocortex. Interestingly, over 70% of 312 neuroscientists believe that long-term memories are maintained by neuronal connectivity and synaptic changes rather than by sub-cellular mechanisms [12]. *But is it really true that the core of memory and consciousness occurs at the level of neural networks and synapses?*

Although neuronal networks/synapses are critical for consciousness and memory, there may be a deeper, sub-cellular level of consciousness and memory that resides “within” neurons—specifically in a neuron’s microtubules (MTs). In possibly acting as mini quantum computers, neuronal microtubules are very stable organelles for memory storage and appear necessary for consciousness [13,14]. An initial model of memory based on MTs was presented by Craddock and colleagues [15] whereby post-synaptic calcium ion flux inward activates calcium calmodulin-dependent kinase II, which then phosphorylates sites on MTs in the post-synaptic neuron to encode memory into MTs. For this model, as well as all theories of consciousness and memory, there appears to be limited, if any, “clinical” evidence that would support a given theory other than possibly EEG and MRI imaging studies [16].

This paper first focuses on consciousness, specifically based on involvement of MTs and their endogenous radiofrequency vibrations for initiating and maintaining consciousness. Two existing quantum theories of consciousness that have a physical basis are presented as the Orchestrated Objective Reduction (Orch OR) theory of Penrose/Hameroff and the Environmental-Induced Decoherence Theory (EID) of Neven. Orch OR advocates that quantum vibrational computations and Superposition Quibits in neuronal MTs produce consciousness. Although Superposition Quibits (SPQs) are central to the EID theory as well, EID postulates that Orch OR’s objective reduction (wave collapse) is not necessary for consciousness. Rather, EID proposes that consciousness is made continuous by the continuous quantum entanglement of SPQs (tubulins). Based on the proposed central role of MT tubules and SPQs in memory, neither theory alone appears sufficient for a quantum explanation of memory. As such, a Hybrid Theory of consciousness (MT/EID) is proposed that involves the continual SPQ formation and SPQ entanglement of the EID Theory, along with MT vibrations of the Orch OR Theory to induce SPQ formation.

Secondly, evidence is then provided that supports two fundamental conclusions on memory: (1) memory resides within neuronal MTs, and (2) radiofrequency vibrations produced by MTs create a microtubule/RF vibrational fabric serving as the basis for memory encoding and consolidation. In contrast to the established view of neuronal circuitry and synapses to explain memory processes, the experimental evidence to be presented is most consistent with refinements to Orchestrated Objective Reduction (Orch OR). Orch OR posits that neuronal microtubules and their vibrations are central to consciousness and memory [13,14,17,18]. Although this paper does not support Orch OR and objective reduction as the primary mechanism for human “consciousness”, it does present modifications to Orch OR as the fundamental sub-cellular process of human “memory”.

Unlike the single state that is “full consciousness”, memory is comprised of two components (short- and long-term memory), both of which must be accommodated in any quantum-based explanation of memory. As such, we present a refinement to Orch OR for memory within MTs that is called “SPQ/OR” wherein: (1) Superposition qubits (SPQs) registering a short-term memory event are in quantum coherence for only a short period of seconds to minutes as short-term memory, (2) Long-term memory consolidation/storage involves SPQs formed for short-term memory of an event being reduced to one of the two tubulin dipole states through objective reduction (OR), and (3) these refinements are based on Quantum Darwinism. Thus, we will posit that memory resides within neuronal MTs through quantum computations made by tubulins. These computations are environmentally modulated by radiofrequency vibrations and photons in the MT’s inner core sea of crystalline water through “Photonic/RF Wave Quantum Computation”. In short, the basis of both short- and long-term memory is proposed in terms of quantum states within MTs—MTs within neurons of a sub-population of brain pyramidal cells in cerebral cortex and hippocampus that specialize in memory [19,20] and are functionally different from pyramidal cells specialized for consciousness [21].

Thirdly, this paper provides evidence that external RF wave treatment to both mice and humans can affect the brain’s MT/endogenous RF wave fabric. The result is enhancement of normal memory in mice and rescue from dysfunctional memory in Alzheimer’s Disease patients. More specifically, studies are presented that forward Transcranial Radiofrequency Wave Treatment (TRFT) as the only MT-targeting agent (MTA) in humans to show clinical efficacy—namely, by stopping and reversing AD cognitive decline [22,23]. The four MT-targeting mechanisms of action provided by TRFT will be described as: (1) disaggregation of toxic oligomers of Aβ and p-tau inside neurons, (2) mitochondrial enhancement, (3) polymerization/repolymerization of MTs, and (4) increasing neuronal activity. Together these TRFT mechanisms stabilize/enhance MT function, resulting in improved memory and even stoppage/reversal of human cognitive decline.

To support the aforementioned tenets for memory, this paper has been structured to initially provide a description of MTs and their endogenous RF waves, then “SPQ/OR” is introduced along with endogenous RF waves and photons as the basis of memory. This is followed by studies showing the ability of exogenous RF wave treatment to enhance normal memory, and finally, RF wave treatment to rescue the dysfunctional memory of Alzheimer’s Disease. It is posited that targeting normal or dysfunctional neuronal MTs with external RF treatment is a successful strategy to enhance normal human memory or rescue human memory impairment (such as that of AD). Moreover, the proposed MT-based actions of RF treatment on neuronal MTs are consistent with the quantum mechanisms of “SPQ/OR”.

Radiofrequency (RF) vibrations/waves, as generated by MTs and as provided externally by TRFT, may be a bridge between the universe and the biology of life. These RF waves, particularly around 1 GHz radiofrequency, seem especially important for memory encoding and processing. Although substantial attention is given to MT involvement in consciousness, this perspective paper focuses on MT involvement in memory because of the empirical evidence we have attained therein. Because both consciousness and memory likely involve MTs, a unified basis for consciousness and memory is provided through neuronal MTs in specifically dedicated populations of cortical and hippocampal pyramidal cells. It should be noted that, although the present quantum-based theory of both consciousness and memory has pre-clinical and clinical support for the “memory” component, the “consciousness” component of this combined theory requires empirical evidence—evidence that should be attainable in future studies.

## 2. Neuronal MTs: Structure, Dynamic Instability, and Memory

**Figure 1:**
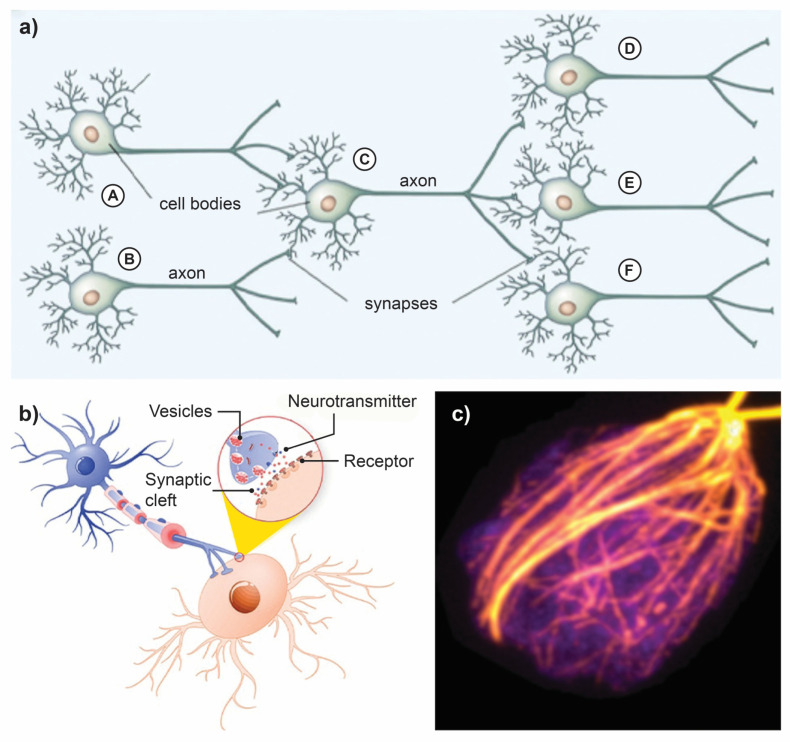
(**a**,**b**) A neuronal circuit and associated synapses with neurotransmitter release, (**c**) A human brain-eating amoeba showing its extensive microtubule network (yellow strands). The six letters in (**a**) refer to each of the six neurons in the neuronal circuit.

Microtubule Structure. Viewing the brain as a computer of simple interconnected neurons and their associated connections (synapses) is the current dogma for explaining memory and cognition (Figure 1a,b). Boosting an impressive 86 billion neurons and 125 trillion synapses, the human brain is indeed a functioning marvel. However, memory and consciousness may not be due simply to connections between neurons because single-cell organisms such as paramecia exhibit complex cognition (e.g., finding food and mates, sex, and learning) without synaptic connections. Rather, such organisms, including brain-eating amoebas (Figure 1c), likely use their cytoskeletal microtubules in these activities [24]. The same microtubules are found inside brain neurons, and their tubulin is the most prevalent protein in the brain.

**Figure 2:**
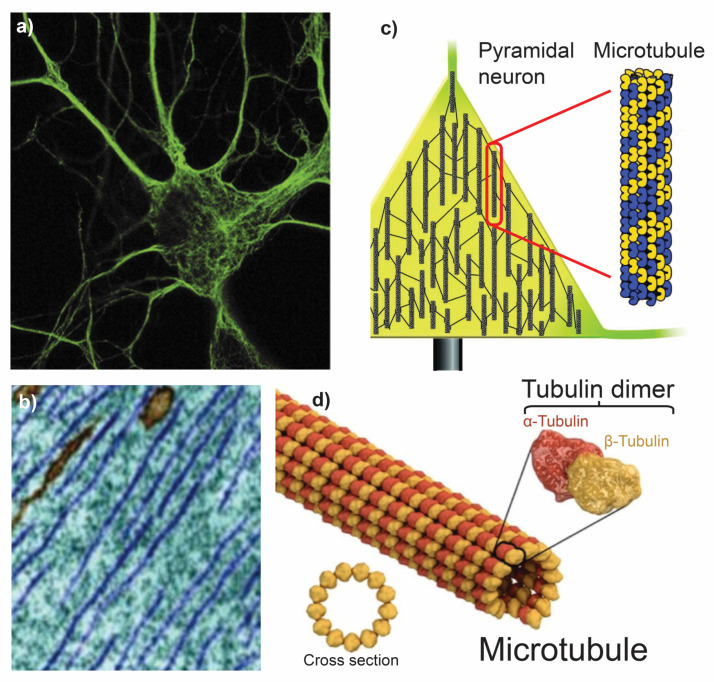
(**a**) A neuron’s MT network shown in green fluorescence, (**b**) Individual intraneuronal MTs, (**c**,**d**) MTs within a cortical pyramidal neuron and the tubulin structure of a MT. (“(**c**)” modified from ref. [14]). In (**c**), individual tubulin dimers of the selected microtubule are shown in blue and yellow.

Microtubules are tubular microorganelles within neurons, each of which is comprised of polymers of the dimer tubulin, with each dimer consisting of closely linked ɑ-tubulin and β-tubulin globular proteins (Figure 2). Each microtubule consists of 13 longitudinal protofilaments made up of polymerized tubulin dimers. Moreover, any given neuronal tubulin is not a single homogenous protein, but rather a complex mosaic of at least 15 different subspecies/isomers that can be phosphorylated [14,25]. Microtubule-associated proteins (MAPs) connect microtubules not only to one another, but to the inner cell membrane as well (Figure 2c). Chief among these MAPs is “tau”, whose phosphorylation is an early causative event in a number of neurodegenerative diseases, including AD [26]. Microtubules have multiple established functions within neurons, including: (1) maintenance of neuronal structure, (2) serving as highways for transporting organelles and proteins down axons to nerve terminals, and (3) involvement in new synaptic formations in dendrites associated with neuronal plasticity. However, there is a proposed function of neuronal microtubules that is often overlooked by neuroscientists, yet this function forms the basis of the present paper—namely, neuronal microtubules being at the sub-cellular core of memory and cognition [13,14].

**Figure 3:**
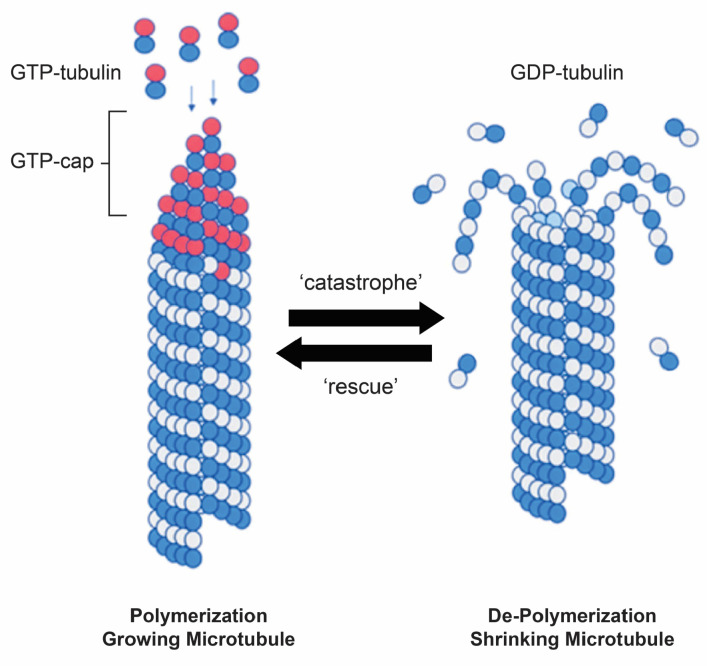
Microtubule dynamic instability between growing (polymerizing) and shrinking (depolymerizing) states. GTP β-tubulin monomers are shown in pink, GDP β-tubulin monomers in gray, and ɑ-tubulin monomers in blue (modified from ref. [28]).

Microtubule Dynamic Instability. MTs undergo both polymerization and depolymerization of tubulin dimers, thus switching between growing and shrinking phases in a process called “dynamic instability” [27,28] (Figure 3). Dynamic instability is characterized by distinct phases of growth and shrinkage. This process occurs exclusively at one pole of a given microtubule. At that pole, the transition from growth to shrinkage (depolymerization of tubulin) is called a “catastrophe”. The reverse transition (polymerization) is called a “rescue”. This process is driven by the binding, hydrolysis, and exchange of GTP on β-tubulin monomers. If the GTP-cap of MT polymerization is lost (low GTP levels), depolymerization occurs or predominates [29].

MTs and MTAs. A long-term balance between growing and shrinking MT states is critical for normal MT function, while a chronic imbalanced state of primarily depolarization (catastrophe) likely results in a variety of neurologic diseases involving memory/cognition, such as Alzheimer’s Disease, Parkinson’s Disease, and a variety of tauopathies (e.g., Frontotemporal Lobe Dementia) [26]. In that context, microtubule stabilization has been shown to be important for memory encoding and consolidation. For example, Uchida et al. [30] found that learning in mice causes biphasic changes in the microtubule networks in their hippocampus in advance of memory consolidation. Specifically, microtubules become unstable 0.5–1 h and hyper-stable 8 h following learning; thus, microtubule stability appears to be involved in memory consolidation.

The aforementioned association of MT depolymerization with a host of neurodegenerative diseases forwarded use of tubulin binders to possibly stabilize MTs. It was thought that, by enhancing MT stability, MT targeting agents (MTAs) would have the potential to restore MT dynamics in these diseases and thus provide therapeutic benefit. Pre-clinical in vitro studies and animal studies with MTAs seemed encouraging for them to treat various neurodegenerative diseases [31]. In two ensuing clinical trials, however, the natural compound epothilone D (EpoD), a potential MT stabilizer, was started in Alzheimer’s patients (NCT 01492374), as was a synthetic MTA agent (TPI-287) in patients with various neurodegenerative diseases (NCT 01966666). Both trials were discontinued because of substantial side effects [28]. As a result, the entire strategy of MTA use and microtubule intervention to treat neurodegenerative diseases was abandoned—that is, until now.

*Section 2 Summary*

- Microtubules (MTs) are intraneuronal organelles made up of tubulin dimers in tubular arrangements of 13 longitudinal protofilaments.
- MTs undergo both polymerization and depolymerization in a dynamic process wherein an excess of depolymerization has been linked to memory/cognitive dysfunction (e.g., Alzheimer’s Disease).
- Use of Microtubule Targeting Agents (MTAs) to stabilize MTs in AD and other neurodegenerative diseases has been unsuccessful.

## 3. Microtubule Vibrations: A Hierarchy of Frequencies

As Stuart Hameroff and Sir Roger Penrose have continued to posit over the past two decades, consciousness is rooted inside neurons, in vibrational states of their microtubules [13,14,17,18,32]. Could the core of human memory also be based on microtubule vibrations and frequencies, as Nikola Tesla famously stated about vibrations and frequencies being key to finding the secrets of the universe? If so, that could explain the failure of previously proposed MTA drugs in clinical trials because they do not impact/modulate MT vibrations.

**Figure 4:**
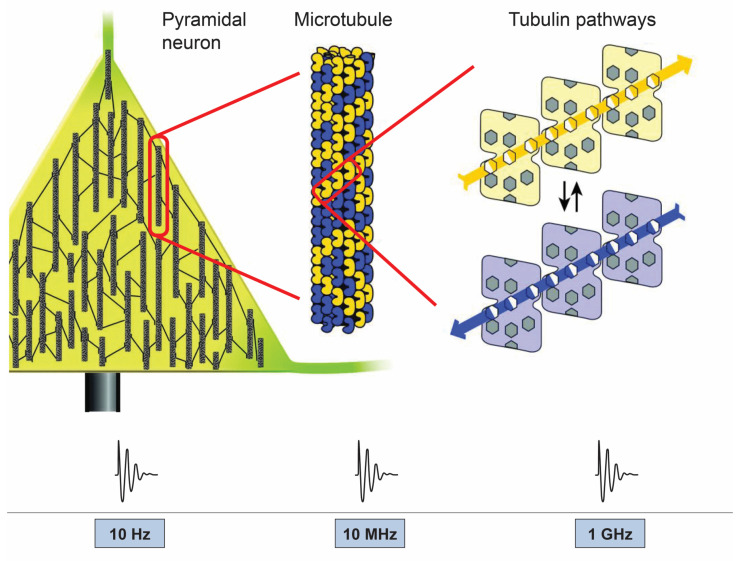
The vibrational range of 10 MHz through 1 GHz encompasses MT and tubulin pathway vibrations. (Right) Three tubulin proteins vibrate and can be in one of two dipole (fixed) states together, as shown, or separately. (Modified from refs. [13,14]).

Order of Vibrational Frequencies in Neurons. For both consciousness and memory, quantum vibrational states in microtubules have been proposed to occur in a microanatomic hierarchy of some 15 orders of vibrational frequencies spanning hertz (Hz), kilohertz (KHz; 10^3^), megahertz (MHz; 10^6^), Gigahertz (GHz; 10^9^), and tetrahertz (THz; 10^12^) frequencies [14]. These vibrations are conceived to be coherent and orchestrated across microtubules, forming a quantum state that produces consciousness or memory encoding events (see Section 4 and Section 5). Central to this order for consciousness and memory is the 10 MHz to 1 GHz range because it involves microtubule vibrations (10 MHz) and tubulin pathway (1 GHz) vibrations (Figure 4). Moreover, this range of vibrations/oscillations is a transition zone between mechano-vibration and radiofrequency vibration [33]. From both pre-clinical and clinical studies (see Section 6 and Section 7), it would appear that the greatest impact on memory processing is around 1 GHz (vibration of tubulin pathways). Parenthetically, higher MT vibration/oscillation frequencies (>1 GHz) may be involved in consciousness, as proposed by Hameroff [14].

**Figure 5:**
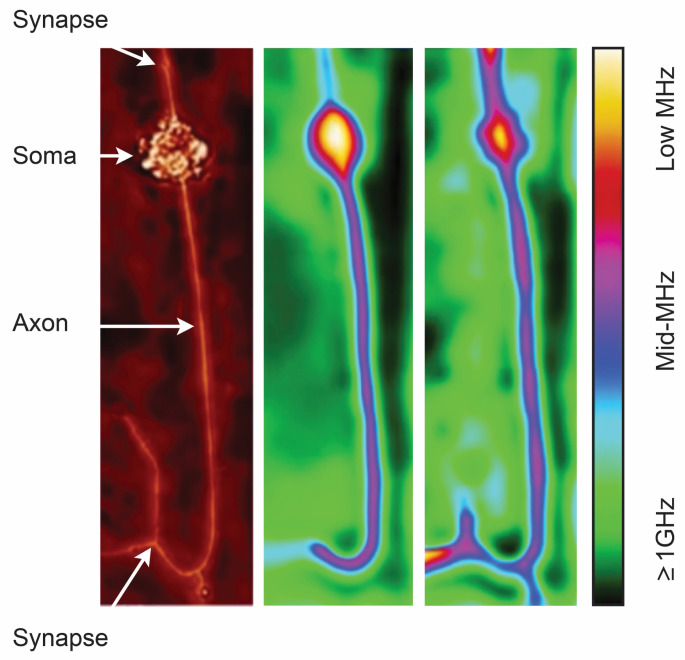
Capture of an immature mouse hippocampal neuron in culture (**left panel**) and its surrounding vibrational/RF fields before firing (**middle panel**) and during a firing event (**right panel**). (Modified from ref. [34]).

In an eloquent series of experiments demonstrating the validity of a microanatomical hierarchy of frequencies, Singh et al. [34] inserted arrays of nanoprobes into the dendrites, somas, and axons of cultured mouse neuronal networks. He and his colleagues detected spontaneous MHz and GHz oscillations from neuronal microtubules before a neuron fires and during a firing event (Figure 5). These endogenous radiofrequency vibrations/oscillations indicate that, in neuronal networks, axonal firings are regulated by deeper, faster radiofrequency vibration activity in microtubules. Moreover, these RF-based connections occur between even physically separated neurons by creating RF fields that pass through gap junctions, connect axonal initial segments with dendrites through the soma, and connect axonal or dendritic branches even when there is no synaptic junction. As such, many RF circuits connecting various components of multiple neurons exist at this deep functional level that appears to be critical for consciousness, memory, and cognition. In a more recent study [35], Singh and colleagues report on their development of an improved EEG device for observing high-frequency brain activity—the Dodecanogram (DDG). The authors report that this 34-electrode head device can sense brain signals in the electromagnetic range of Hz to THz. If so, DDG may have the potential to identify intraneuronal structures (such as MTs) vibrating faster than neuronal membranes that could not otherwise be sensed by EEG. Although DDG electrodes on the scalp may be sensitive enough for detecting brain activity in the important 10 MHz to 1 GHz range indicated previously, the ability of higher frequencies (generally greater than 5 GHz) to penetrate the human cranium is questionable. At least through 1 GHz, the DDG device could be of substantial value in detecting high-frequency brain activity related to cognitive activity. The work of Singh et al. [34,35] clearly advocates that histologically viewed neural circuits and their synapses are only a partial and more superficial view of the many deeper-operating RF circuits in neural networks.

As discussed in Section 6, exogenous RF waves at 1 GHz can enhance cognition most likely by modulating these complex and deeper-operating RF circuits.

Vibration of MT Tubulin Walls and Central Water Cores. MTs are polar and electric dipoles, whose tubulin walls typically vibrate/oscillate between 10 and 100 MHz [36,37]. All 13 component protofilaments of a MT vibrate in unison within this range, although peak mechanical vibration can occur at 1500 MHz [38]. The energy for these vibrations comes from GTP and ATP produced by nearby mitochondria—thus, mitochondria are the source of energy for a neuron’s vibrational/RF wave generation. These intra-neuronal vibrational systems in turn produce both an RF field and an electric field within and around the MT, which are relatively small [33]. Additionally, microtubule-associated proteins (MAPs) such as tau interconnect MTs to create a common vibrational system (Figure 4).

The highest vibrational frequency of neuronal microtubules is around 1 GHz [36,39]. However, MT cores are filled with crystalline water that vibrates at or above 1 GHz [40]. At and around 1 GHz, vibrations are a critical frequency range where both the microtubule protein lattice and its internal crystalline water structure may interact dynamically. Moreover, this water core integrates all tubulin proteins in MT walls such that the MT, irrespective of its size, can function like a single protein molecule [33] to induce coherent MT oscillations. As such, the crystalline water core of an MT controls the functions of that MT [40,41]. The transition from independent tubulin protein vibrations to synchronized (coherent) vibrations of an entire MT occurs due to crystalline water vibrations and the energy provided by GTP hydrolysis to MT walls, such that a critical threshold is reached for the MT to act as a single, large-scale dipolar protein [33]. Since vibrations at or above 1 GHz occur in MT inner water cores [40], administration of RF waves at 1 GHz would be expected to vibrate MT inner water cores (in linked resonance/dipole coherence) to modulate function of the entire MT. Most recently, Hameroff and colleagues [42] have hypothesized that neuronal microtubules behave as “fractal time crystals” characterized by resonance oscillations in a hierarchy of similar dynamics that repeat in hertz, kilohertz, megahertz, gigahertz, and terahertz frequencies [42]. As discussed in the next section, the vibration of microtubules in Layer 5 pyramidal neurons of the cerebral cortex appears to be involved with initiating and maintaining consciousness [14,21].

*Section 3 Summary*

- Although there is a hierarchy of possible vibration frequencies in neurons, the frequencies between 10 MHz and 1 GHz appear to be particularly important because they involve whole MTs, their component tubulin pathways, and likely the MT inner water core to induce coherent MT oscillations.
- Vibrations spontaneously occur in the low MHz to 1 GHz+ frequency range within and around neurons, with MT vibrations occurring before and during neuronal activation.
- Mitochondria provide the energy for MT vibrations, while the crystalline water core of MTs controls MT function.

## 4. “Quantum Consciousness” Through Vibration and Continual Formation/Entanglement of Superposition Qubits Within Neuronal Microtubules of Consciousness-Dedicated Neurons

**Figure 6:**
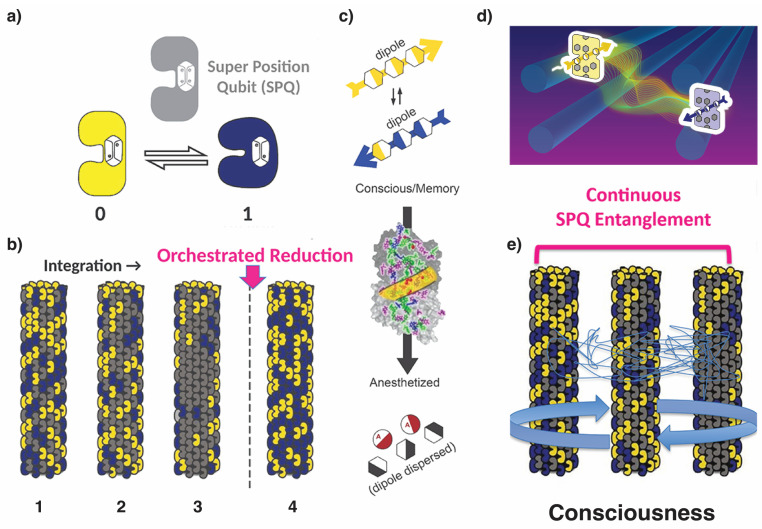
(**a**) Tubulin dimers can exist in one of two dipole states (yellow, blue) or in both states simultaneously (gray), (**b**) In Orch OR theory, a “conscious” event by quantum MT computing occurs by superposition (gray) tubulins increasing in number during an integration phase (1–3), followed by a reduction event of Orchestrated Reduction (4) to produce a consciousness moment, (**c**) The region of tubulin containing inner hydrophobic aromatic rings that gas anesthetics bind to (yellow band) for induction of dipole dispersion (anesthesia), (**d**) Four MTs, showing two SPQs in two different MTs in quantum entanglement, (**e**) In the proposed Hybrid Theory, a conscious moment occurs when an SPQ forms and consciousness is made continuous by the constant circling of SPQ quantum formation and entanglement. (**a**–**c**,**e**) modified from refs. [13,14].

In 1989, Sir Roger Penrose proposed that something analogous to “quantum computing” occurs in the brain, but that for conscious processes, this computing would need to terminate in accordance with self-collapse by a form of non-computable objective reduction [43]. This led to a faithful collaboration between Penrose and Stuart Hameroff that resulted in the concept of Orchestrated Objective Reduction (Orch OR) to explain consciousness [13,14]. Orch OR does not involve conformational changes in MTs or their component tubulins, but rather in tubulin dipole states that then represent consciousness/information [44]. Tubulin can exist in one of two dipole states or in both states (superposition qubits; SPQs) simultaneously (Figure 6a). According to the Orch OR theory of consciousness (Figure 6b), quantum superposition qubits increase in number during integration phases (1–3) until threshold is met for a conscious moment. At this time, Objective Reduction occurs wherein SPQs select one or the other dipole state, thus providing for a consciousness moment (phase 4). Hameroff has proposed that this sequence repeats many times per second (around 40/s) to provide consciousness, much like the sequence of frames in a movie reel provides motion in a movie. Thus, the Orch OR theory proposes that consciousness occurs well below the level of neuronal circuits and their synapses in MTs, and appropriately dubbed “the quantum underground” [45].

Critical to Orch OR is that MT vibrations (see Section 3) induce the formation of super position qubits (SPQs) that then produce consciousness. For this and other quantum theories of consciousness, it is important to first discuss how MT quantum vibrations and formation of SPQs might induce consciousness at a level below that of neuronal activity and neural networks. Although neuronal activity “orchestrated” by synaptic inputs impacts MTs in post-synaptic neurons, MT vibrations in those neurons subsequently regulate slower membrane and synaptic activities within their post-synaptic resident neuron [34]. Thus, Orch OR takes into account the important ability of MTs to vibrate (especially between 10 MHz and 1 GHz) to induce coherent vibrations/oscillations of the entire MT (see previous section) to then induce SPQ formation, orchestrated reduction, and resulting consciousness. The energy for such MT vibrations comes from GTP, with the MT crystalline inner water core (next section) acting as a waveguide to synchronize all tubulins for in-phase (coherent) vibrations.

The proposed critical nature of MT involvement in consciousness is illustrated by the mechanism of action for gas anesthetics. For many years, it was thought that gas anesthetics acted as GABA receptor agonists and thus exerted their anesthetic ability outside of neurons on neuronal surface membranes. In the 1980s, however, Hameroff and Watt argued that dipoles in intra-protein hydrophobic regions of neurons coupled and oscillated coherently, and that this coupling was necessary for conscious awareness [46]. They further suggested that anesthetic gases bind in these non-polar, hydrophobic regions to disperse endogenous coherent dipoles necessary for consciousness. More recently, computer modeling has been used to demonstrate anesthetic binding sites in tubulin’s hydrophobic channels of non-polar aromatic rings (Figure 6c) [32,47]. Thus, it would seem that dipole dispersion/incoherence in neuronal microtubules is the most logical mechanism for gas anesthetic actions in going from full consciousness to unconsciousness.

Contrary to Penrose and Hameroff’s Orch OR, Neven and colleagues [48] have recently proposed that consciousness arises whenever quantum superpositions form, which is continuously during consciousness. Thus, they posit that orchestrated reduction (wave collapse) is not necessary for consciousness. Rather, that consciousness is made continuous by always-forming SPQs continuously entering into quantum entanglement with other SPQs within MTs of those neurons and other neurons. (Figure 6d,e). In other words, they have proposed that a conscious moment occurs whenever a given superposition forms, and that there is no collapse of the wave function (i.e., no objective reduction). Another difference with Orch OR is that there is no mention of MT vibrations in Neven’s quantum theory. Neven and colleagues [48] believe that quantum entanglement of quantum superpositions naturally solves any binding problem, which would then ensure a continuum and unity of consciousness. Thus, Neven and colleagues [48] propose that, at any given moment, a human can only experience one classical state. As such, when a new superposition forms in MTs and entangles with other superpositions, we experience classical basic states that result in distinct sequences of conscious moments. This is essentially a “collapse” since a given quantum system (SPQs) has interacted (entangled with) the environment (other SPQs) to effectively cause Environmental-Induced Decoherence (EID); in essence, this interaction appears as a collapse of the superposition.

In evaluating both the Orch OR theory and the new theory of EID, *a Hybrid Theory of consciousness, comprised of components from both quantum theories, would appear to be most viable and is proposed.* This Hybrid Theory (MT/EID) involves the MT vibrations and coherent oscillations of the Orch OR Theory, along with the continual SPQ formation and SPQ entanglement of the EID Theory. Orch OR’s objective reduction (collapse of the waveform) is not included in this Hybrid Theory because the system of SPQs themselves continuously collapse due to EID. Along that line, entanglement of SPQs is central to the EID theory [48] and the current Hybrid Theory (MT/EID), but is only discussed in general terms in Orch OR—usually in reference to quantum computation and infrequently in association with SPQs [13,14]. In emphasizing the entanglement of SPQs, this MT/EID Theory negates the issue of when a measurement occurs because it is continuous, with continual formation and entanglement of SPQs. Thus, for multiple reasons, the Hybrid (MT/EID) Theory fits best into our unifying view of memory.

**Figure 7:**
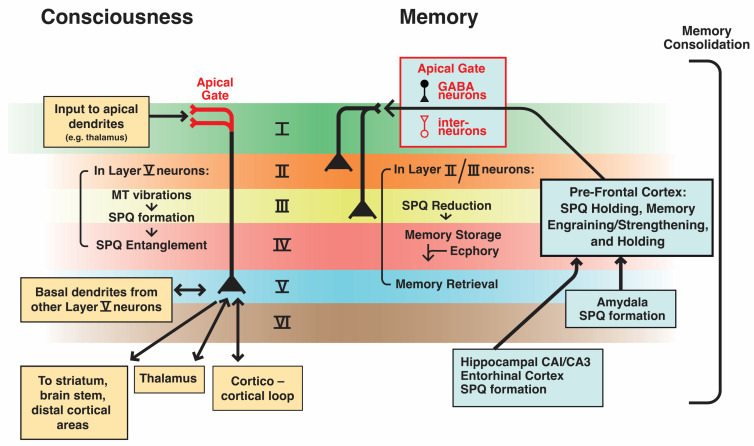
(**Left**) The MT/EID Hybrid Theory of consciousness involves the MT vibrations and coherent oscillations of the Orch OR Theory, along with the continual SPQ formation and SPQ entanglement of the EID Theory. Apical dendrites of cortical Layer V pyramidal neurons in cortical Layer I serve as an “Apical Gate” for consciousness initiation in pyramidal neurons. (**Right**) The SPQ/OR Theory of memory involves SPQ formation (short-term memory) in multiple brain areas, with memory engraining/strengthening and holding in the Pre-Frontal Cortex. Following processing through an “Apical Gate”, future long-term memory is processed through dendrites of Layer V neurons in cortical Layer I, followed by SPQ reduction, memory storage, and later memory retrieval primarily through Layer V pyramidal cells. Note: Only primary neurons/connections proposed to be involved in consciousness and memory are shown.

It is important to recognize that recent transcriptomic studies have found dozens of pyramidal cell subtypes in the human brain based on anatomic morphology/projections, gene expression, and functional differences [49,50]. This diversity of pyramidal cells encompasses specialized roles of some pyramidal cells in Layer V of the cortex in conscious processing, while other pyramidal cells in both hippocampus and superficial cortical layers II and III are critical for memory processing (see next Section 5) [19,21]. Thus, as currently proposed, our Hybrid Theory involves “consciousness”—dedicated pyramidal cells primarily in Layer V of the cerebral cortex (Figure 7, left) [21]. Layer 5 pyramidal cells have extensive dendritic trees extending up to Layer 1 of the cortex, which are important for the integration of incoming information related to consciousness (Figure 7, left) [14,51]. Indeed, a specific terminal region of these apical dendrites of consciousness-dedicated pyramidal cells acts as a gate or switch for consciousness [21]. In keeping with our present quantum concept of consciousness, these apical “gate” dendrites of Layer V pyramidal cells likely have an internal threshold level of MT vibration, SPQ formation, and/or SPQ entanglement for activating consciousness in Layer V neurons. Important for activation of apical gate dendrites is direct input from the intralaminar and medial thalamic nuclei (Figure 7, left) [52]. This thalamic-to-cortex input helps bring Layer V neurons to an elevated state necessary for initiation and maintenance of consciousness [53]. As such, *dipole coherence in MTs of Layer V pyramidal cells (likely in apical gate dendrites) is proposed to initiate consciousness. Conversely, dipole incoherence in MTs residing in these apical dendrites is posited to initiate and promote sleep.*

In addition to their apical dendrites, Layer V pyramidal cells have an extensive network of basal dendrites that serve to communicate with basal dendrites in other pyramidal cells, thus forming a network of pyramidal cell basalar dendrites (Figure 7 left) [54]—an anatomic net covering the entire brain that fits well with SPQ formation and entanglement in Layer V pyramidal cells as central to neuronal coherence and resulting consciousness. Thalamic input to these basilar dendrites has been proposed to amplify the Layer V signal for consciousness, thus augmenting broadcast of consciousness across the broad network of pyramidal cell basilar dendrites.

Outflow (efferent) evidence of these Layer V pyramidal cells as being critical for consciousness involves them being at the nexus of both thalamo-cortical and cortico-cortical loops (Figure 7 left) [55,56]. More specifically, Layer 5 consciousness-dedicated neurons project to higher-order thalamus areas in an important two-way thalamo-cortical loop for consciousness, as well as to striatum, brain stem, and distal cortical areas [21]. With all of their afferent and efferent connections with other neurons, these Layer V consciousness-dedicated neurons are ideal for being the central nexus of consciousness. Moreover, MTs and their component tubulins within these large Layer V pyramidal cells vibrate, most relevantly in the 10 MHz–1 GHz range for our Hybrid Theory (Figure 4) [14,37]. It is important to note that, although the cerebral cortex and Layer V pyramidal cells are required for complex thoughts and language, even the absence or complete removal of the human cerebral cortex still results in a basic state of consciousness (e.g., feeling emotions, reacting to one’s surroundings, waking up) due primarily to structures in the brain stem [57].

At this point, it may be helpful to compare our MT/EID quantum theory of consciousness with the three prominent theories of consciousness (IIT, GWT, and GNWT) described in the first paragraph of Section 1 [1,2]. The currently-presented MT/EID quantum theory of consciousness would appear to have design advantages over these more established theories—namely that MT/EID provides for specific consciousness organization at all three anatomic levels—brain area level, neuronal pathway/network level, and neuronal micro-structural (microtubule) level. By contrast, Integrated Information Theory (IIT) does not define consciousness based on specific anatomical brain areas, neuronal pathways, or sub-cellular mechanisms. Instead, it begins with fundamental axioms of subjective experience and mathematically derives the properties that any physical system must possess to be conscious [58]. So rather than providing a rigid anatomical map, IIT uses a mathematical framework to evaluate whether *any* physical substrate’s causal interactions are sufficiently integrated and irreducible. The Global Workspace Theory (GWT) does not specify brain areas or neuronal pathways. However, its biological extension, the Global Neuronal Workspace Theory (GNWT), specifies the prefrontal cortex, parietal cortex, and anterior cingulate gyrus as central hubs of consciousness. In the GNWT theory, consciousness relies on a massive web of long-range cortico-cortical connections (Layers II/III and V/VI) and thalamocortical loops [1,59]. For consciousness, these pathways must possess enough strength to trigger the prefrontal cortex to broadcast the representation throughout the brain.

Neither the IIT nor the GWT has enough physical detail at any of the aforementioned three anatomic levels to compare with our MT/EID quantum theory. The GNWT does have some brain areas and neurons (e.g., Layer V pyramidal cells) in common with the MT/EID theory, but has a different pathway for consciousness activation (via PFC) compared with MTs/EID. Moreover, it does not have a clearly defined sub-neuronal mechanism of action beyond activation of neural networks [1], while the quantum MT/EID theory has a clear sub-neuronal mechanism of microtubule action for consciousness. Thus, it would appear that the MT/EID theory has some commonality with the GNWT, but not much. Indeed, the MT/EDI theory’s encompassing and explicit “tri-level” explanation of consciousness should be eminently testable in future studies. For example, the MT/EID would predict that cortical Layer V would be much more active in brain imaging (fMRI, FDG-PET) during consciousness compared to sleep due to its pyramidal cells central in the MT/EDI theory.

In addition to the MT/EID quantum theory of consciousness presented in this section, the next section presents our quantum MT theory of memory involving a connectivity- different and functionally-different population of “memory-dedicated” pyramidal cells residing in both hippocampus and neocortex. Together, these similarly based theories form a unifying concept involving MTs, their vibrations, and resultant SPQ formation in providing for consciousness and memory.

*Section 4 Summary*

- Two quantum theories of consciousness are the Orchestrated Objective Reduction (Orch OR) theory of Penrose/Hameroff and the Environmental-Induced Decoherence Theory (EID) theory of Neven.
- Although Superposition Quibits (SPQs) are central to both theories, the EID theory postulates that Orch OR’s objective reduction (wave collapse) is not necessary for consciousness. Rather, EID proposes that consciousness is made continuous by the continuous quantum entanglement of superposition tubulins.
- A Hybrid (MT/EID) Theory of consciousness is proposed that advocates cortical Layer 5 “consciousness-dedicated” neurons as the central nexus for consciousness. Within these consciousness-dedicated neurons, continual formation of SPQs and SPQ entanglement of the EID Theory occurs, along with the Orch OR Theory’s MT vibration induction of SPQ formation. This Hybrid (MT/EID) Theory fits well with our unifying view of both consciousness and memory being presented in this paper.

## 5. Proposed Short-Term and Long-Term Memory: Quantum Computing by Neuronal Microtubules via Super-Position Qubits and Orchestrated Objective Reduction (SPQ/OR)

**Figure 8:**
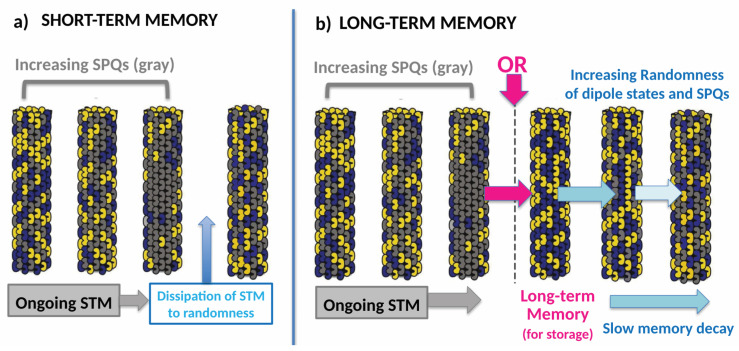
(**a**) The process of “short-term memory” in neuronal MTs is proposed to be brought about by increasing numbers of Superposition Qubits (SPQs), with dissipation to randomness within minutes. (**b**) By contrast, for “long-term memory”, SPQs in MTs are posited to be reduced to one of the two tubulin dipole states in Objective Reduction (OR). If the memory is not to be permanent, a progressive, slow decay would occur that likely involves conversion of tubulin dimers into progressively more random dipole states and random SPQs.

It is important to recognize that “quantum consciousness” differs from “quantum memory” in that “full consciousness” is essentially a single classical state—either you are fully conscious or you are not. Parenthetically, the author and others consider lucid dreaming and controlled dreaming to be intermediate states between full consciousness and unconsciousness [60,61]. On the other hand, memory is basically comprised of two primary components: Short-term memory (over seconds to a few minutes with fast decay) and Long-term memory (hours to permanent with slow/no decay). As such, if MTs are involved with memory, they must account for both short- and long-term memory. The following proposed refinement of Orch OR can accommodate both major components of memory through neuronal MTs. In short-term memory (Figure 8a), quantum superposition qubits in MTs would increase in number during integration phases (1–3) similar to Orch OR—but without Orch OR’s “objective reduction” (Figure 6b). In other words, it is proposed that *the process of short-term memory in neuronal MTs can be completely accommodated by an increasing number of Superposition Qubits (SPQs) (*Figure 8a). It is then posited that, for any short-term memory event in a neuron’s MTs, SPQs register that event for a short period of seconds to minutes. During that period, progressive decay of SPQs occurs, with decay (i.e., loss of the short-term memory event) within a few minutes. This is in keeping with SPQs for any given short-term memory event being transitory by its very nature. As primary brain areas involved in formation of short-term memories, the hippocampus, associated entorhinal cortex, and amygdala are proposed to be the main areas where neuronal MTs form new short-term memories (Figure 7, right).

For longer-term/permanent memory of the event (Figure 8b), SPQs within MTs of short-term memory-based neurons would need to be integrated and reduced to one of the two tubulin dipole states through objective reduction (OR). SPQs formed in the hippocampus/entorhinal cortex and amygdala would presumably then be consolidated (stabilized and strengthened) into “engram memory” bit formations primarily in the prefrontal cortex (PFC; Figure 7, right). Such new memories would then be held in the prefrontal cortex before being sent to the cerebral cortex for storage and later retrieval (Figure 7, right). As such, memory could be stored in the cerebral cortex within neuronal MTs as a series of tubulins in one dipole state (such as 111) or in a combination of dipole states (such as 010). Thus, chains of tubulins, all in one dipole position or the other, or in a combination of dipole positions, could provide for memory storage within neuronal MTs of the cerebral cortex. The aforementioned refinement of Orch OR to SPQ/OR posits that OR occurs for long-term memory at some point between short-term memory areas (e.g., prefrontal cortex) and storage in cerebral cortex pyramidal cells—most likely within dendrites and cell bodies of those cells. 

It is important to indicate that, unlike the proposed entanglement of SPQs between many largely Layer V pyramidal cells to provide a global cortical network of consciousness (Figure 7, left), it is postulated that quantum memory involves only limited or no entanglement of SPQs within neurons or between neurons (Figure 8). Rather, we propose it is a more localized quantum process that ultimately involves “localized” orchestrated reduction (collapse of the wave form) to engrain long-term memory traces within small groups of neurons. This is very similar to the small subset of pyramidal neurons called “engram cells” that are proposed to store and retrieve memory events in the Engram Cell Theory of memory [9] and anatomically consistent with “cortical minicolumns”, which are the smallest functional units of the cortex [62] (see below).

Since MT-based memory storage would be very stable over time, it should be easily retrievable. However, for “weak or modest” long-term memories being stored in the cerebral cortex (or elsewhere) that dissipate over hours/days, this progressive memory loss of a stored memory event would likely be due to MT tubulins progressively going from one of the two tubulin dipole states to a progressively random display of SPQs and dipole states. Thus, some long-term memories stay around for years (no conversion to SPQs), while others dissipate over days/months (conversion from a dipole state to randomness). In contrast to the aforementioned objective collapse of SPQs in OR to explain long-term memory, it is difficult to envision how the Environmental-Induced Decoherence (EID) theory of Neven and colleagues could accommodate long-term memory; this is because it denies such objective collapse by proposing branching realities instead [48]. As such, only quantum long-term memory based on the Orch OR theory is proposed to occur.

It should be underscored that there is evidence for specific pyramidal cells in cerebral cortex and hippocampus dedicated to memory and that they are quite different in their “connections” and “functionally” from the previously-described Layer V pyramidal neurons in cerebral cortex whose function is critical for consciousness (see Section 4) (Figure 7, right) [19,21]. More specifically, it is proposed that hippocampal CA1 and CA3 neurons, entorhinal cortex (ENT), and amygdala are involved in initial SPQ formation (memory encoding) of an event, with stabilization and strengthening of such memory encoding primarily in the PFC. The PFC is where such memory tracing would be temporarily held, thus being the main hub for keeping short-term memories accessible (Figure 7, right). *To summarize, we propose that initial encoding/consolidation of short-term memory occurs through an increasing number of SPQs being formed for the event in hippocampus/ENT/PFC.* Likewise, utilizing the same increase in SPQ encoding, the amygdala would handle initial emotional memories, which are then held in the PFC.

If an event is to be incorporated into long-term memory storage, the PFC would relay this information to cortical Layer I, which acts as a “gateway” to long-term memory [63] via apical synapses with memory-dedicated pyramidal cells located primarily in cortical Layers II and III (Figure 7, right). Almost all of the few neurons in Layer I are GABAergic cells. It has been proposed that these GABAergic neurons and Layer I interneurons play a critical role in cortical processing in that they can “gate” (inhibit or disinhibit) information from other cortical areas (e.g., PFC) that carry cognitive information to apical dendrites of Layer II/III pyramidal neurons in Layer I [63]. Through the process of memory consolidation, Layer II/III pyramidal cells are likely critical for memory storage and retrieval across multiple cognitive domains. This has been proposed to occur through specific “memory engram” neurons within Layers II/III [64]. It is further proposed that *the transfer of short-term memory occurs across the synaptic input to Layer II/III pyramidal cells via both synaptic activity and RF wave activity. Memory engraming would then occur through “localized” SPQs within MTs of Layer II/III pyramidal cells being reduced to one of the two tubulin dipole states through Objective Reduction*, as previously discussed (Figure 6).

As briefly mentioned earlier in this section, it should be noted that cortical pyramidal cells are anatomically arranged into “cortical minicolumns”, which are the smallest functional units of the cortex [62]. Each minicolumn extends through all six cortical layers and is approximately 50 μm wide, with each minicolumn consisting primarily (80%) of Layer II, III, and V pyramidal cells [65]. Although the Layer V pyramidal cells in each minicolumn appear to be largely involved in consciousness (see Section 4), the Layer II/III pyramidal cells within each minicolumn are principally involved in memory/cognition within a localized “minicolumn” environment [65]. As mentioned earlier in the Engram Cell Theory of memory [9], the subset of pyramidal cells in Layers II/III called “engram cells” could store and retrieve memory events [9]. Regarding memory retrieval, it has been generally established that retrieval involves an interaction between external sensory/internal cues and stored memory traces/engrams [66]. This process is termed “Ecphory” or a reawakening of memory traces/engrams. Parenthetically, engrams have not yet been identified empirically. Thus, memory retrieval is best understood as cue-induced expression of the engram. Both the concept of localized cortical minicolumns and localized engram cells dedicated to specific memory events support our view that memory consolidation in the cerebral cortex likely involves “localized” SPQ reduction in pyramidal cells located primarily within cortical Layers II/III.

MTs in dendrites are very stable and can remain assembled indefinitely because they are capped by MAPs that prevent depolymerization [67]. Since MTs in “dendrites” are in close apposition to pre-synaptic nerve terminals and synapses with incoming information, they are ideally positioned to process memories. As such, the MTs in dendrites/dendritic spines are thought to be most involved in “quantum computing” [13,14]. Moreover, once neurons mature, they do not divide [68], so neuronal MTs can remain safely assembled indefinitely, perhaps for the lifetime of an individual. Each microtubule is potentially able to encode vast amounts of information. In this regard, each microtubule has the potential to do 10^16^ functions/second—this is the same as for all synapses of all neurons in the entire human brain [14]. It should be apparent that much more information could be stored when the human brain is seen from a MT-based memory perspective. Perhaps reassuring to humans, the memory-based activity of MTs in all of the brain’s neurons, along with their associated endogenous RF vibrations/oscillations, would be extraordinarily difficult (conceivably impossible) for AI to integrate into any “synthetic brain” with both intellect and consciousness.

The SPQ/OR theory posits MTs as central to memory through their multiple orders of vibrational frequency, particularly within the radiofrequency range of 10 MHz–1 GHz. This suggests that administration of external RF waves could have medical applications by addressing the marked microtubule dysfunction in various neurodegenerative diseases [69]. As will be discussed in detail in Section 8, Alzheimer’s Disease is profoundly impacted by a destabilization of neuronal MT structure, resulting in MT depolymerization and ensuing memory loss.

*Section 5 Summary*

- Orchestrated Objective Reduction (Orch OR) states that quantum vibrational computations in neuronal MTs are “orchestrated” with synaptic input to produce consciousness.
- Since memory is comprised of two primary components (short- and long-term memory), a refinement of Orch OR within MTs called “SPQ/OR” is proposed wherein: (1) Superposition qubits (SPQs) registering an event are present for a short period of seconds to minutes as short-term memory, and (2) SPQs for short-term memory of an event are reduced to one of the two tubulin dipole states through objective reduction (OR) to consolidate long-term memory. According to the SPQ/OR theory, quantum short-term memory encoding occurs primarily in hippocampus/entorhinal cortex pyramidal cells and is strengthened in prefrontal cortex, while quantum long-term memory storage and retrieval occur primarily in cortical Layer II/III pyramidal cells.
- Microtubule vibrational frequencies, particularly within the RF range of 10 MHz–1 GHz, advocate that administration of external RF waves within this range could enhance MT function or correct MT dysfunction

## 6. “External” RF Waves, Photonic/RF Wave Quantum Computation, and Quantum Darwinism

**Figure 9:**
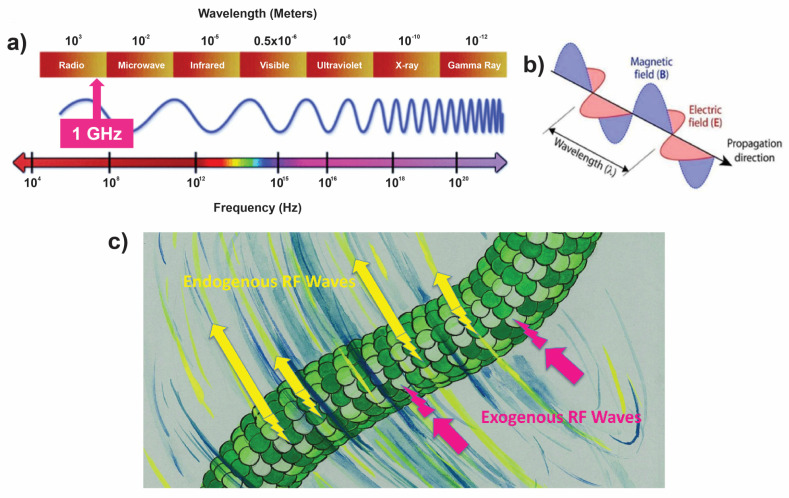
(**a**) RF waves are at the lower end of the electromagnetic wave spectrum, with 1 GHz of particular importance inthis paper, (**b**) An RF wave consists of linked sinusoidal electric and magnetic waves, (**c**) A MT’s internally-produced RF waves (yellow) can affect that MT and its proximal surrounding, while “exogenous” RF waves (pink) waves can affect all MTs within any given neuron of the human brain.

Radiofrequency (RF) waves are within the electromagnetic wave spectrum as the waves of lowest frequency range—we define RF waves (UHF) as between 0.3 and 2.0 GHz, with 0.9 GHz–1.0 GHz designated as 1 GHz) (Figure 9a) RF waves, as with all electromagnetic waves, consist of linked sinusoidal electric and magnetic waves that together generate an electric field, magnetic field, and direction of wave propagation all aligned perpendicular to each other (Figure 9b). In a given neuronal MT, internally produced vibrations can affect that microtubule’s MT vibrational fabric (Figure 9c). Extending this premise, it is proposed that “exogenous” (treatment-based) RF waves can beneficially affect this MT vibrational fabric within MTs and do so through a refinement of Orch OC with inclusion of Quantum Darwinism.

**Figure 10:**
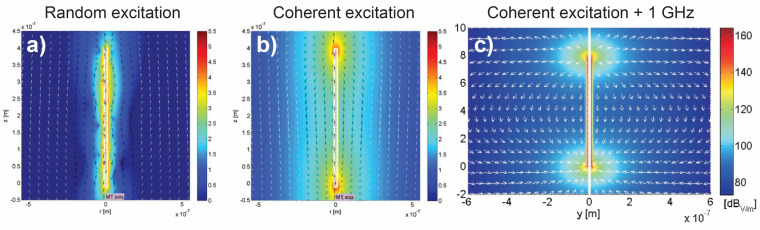
(**a**) Uniform electric field generated by an MT with random tubulin dipoles, (**b**) Enhanced electric fields at MT poles with all of its tubulin dipoles in phase, (**c**) In an MT with its tubulin dipoles in phase, a 1 GHz external RF field generates a 30-fold increase in electric field at its poles. (Modified from refs. [70,71]).

As demonstrated by Singh et al. [34] endogenous RF waves are far faster than synaptic activity and thus are critical to memory cognition. Two related concepts are: (1) Microtubule vibrations/RF fields can be greatly increased by external RF wave application, and (2) There is an externally applied radiofrequency that may be optimal for enhancing human memory/cognition. Regarding the first concept, Havelka and colleagues [70,71] performed computer simulations of single microtubules and the electric fields they generate. When the oscillation of all tubulin dipoles was random, the electric field distribution was uniform along the length of the microtubule (Figure 10a). However, when all tubulin dipoles were in phase with coherent oscillation, the microtubule acted as a single dipole with enhanced electric fields at its poles (Figure 10b). Moreover, this polar increase in electric fields generated by MTs in phase was greatly increased 30-fold by external RF waves at 1 GHz frequency (Figure 10c) [71]. Thus, although each tubulin has its own dipole, RF stimulations result in the MT acting as one giant dipole, whose electric field can be greatly enhanced by external RF waves at 1 GHz.

In addition to the aforementioned computer simulation and cell culture studies showing beneficial biophysic/physiologic effects of 1 GHz application, it is proposed that “externally administered” 1 GHz is at or near the optimal radiofrequency for benefiting “memory/cognition”. Multiple studies are supportive of this conclusion. First, in both mice and humans, 1 GHz RF waves improve memory [22,23]. Second, radiofrequency waves around 1 GHz increase EEG alpha wave activity, which facilitates memory processing [72,73]. Third, 1 GHz radiofrequency waves stabilize/repair microtubules by suppressing p-tau formation and aggregation [23]. And fourth, all MT microfibrils usually vibrate in unison at 5–100 MHz, so applying a higher external frequency (1 GHz) should enhance MT vibration above normal [33,36,37,38]. Peak vibration of MTs occurs at 1.5 GHz [38], so 1 GHz would appear to be ideal for memory enhancement. Our experimental support for the beneficial use of RF waves at 1 GHz for memory/cognition will be detailed in succeeding Section 7 and Section 8.

**Figure 11:**
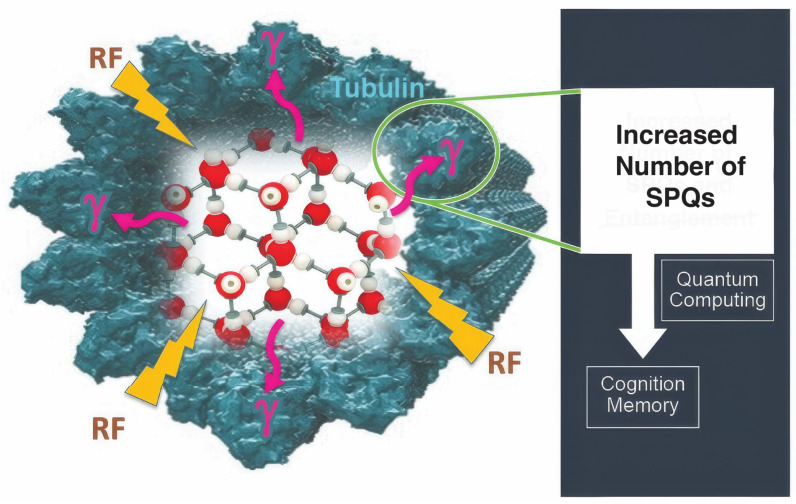
Crystalline water fills the inner core of a microtubule. Both internally and externally generated radiofrequency waves (yellow bolts) can excite the water core. Excitation induces the water core to generate photons (pink arrows) and/or radiofrequency waves that can then interact with tubulin qubits in microtubule walls to induce quantum coherence across the microtubule through increased SPQ formation. Quantum computing would then result, with consequent memory processing.

**Figure 12:**
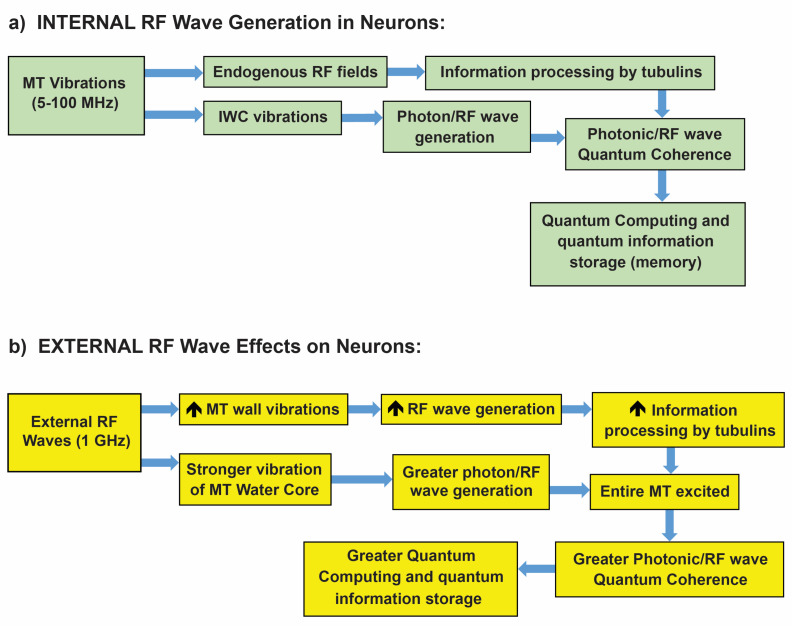
A summary of proposed internal (**a**) and external RF wave effects (**b**) on MTs to produce photonic/RF wave computation. Upward arrows in (**b**) indicate an increase compared to Internal RF Wave generation alone.

Photonic/RF Wave Quantum Computation. The inner core of MTs is filled with “crystalline” water, which we propose to play a critical role in memory processing by MTs (Figure 11). This inner water core (IWC) of latticed water molecules vibrates at a higher frequency than MT microfibrils (at least 1 GHz). So IWC vibrations coordinate and integrate the entire MT to vibrate/function as a single protein molecule [40,41]. “Internal” RF waves can enter the crystalline IWC, resulting in a higher energy state (then lower state) for water, generating subliminal photons (biophotons) and/or RF waves (Figure 11) [74]. These induced photons/RF waves can then interact with tubulin qubits in MT walls to provide for quantum coherence across the MT through increased SPQ formation (Figure 8a,b and Figure 11). The result is *“Photonic/RF Wave Quantum Computation”* and consequent memory encoding and consolidation as shown in Figure 12a.

In reference to the aforementioned beneficial effects of 1 GHz application on memory, a frequency of 1 GHz is common in quantum computing since it is within the RF range where superconducting qubits can be controlled. It cannot presently be indicated whether photons or RF waves emanating from the IWC are more important for memory. Inclusion of RF waves with photons as emanating from excited IWCs to affect memory is based on the passage of RF waves through any material, including ice, which excites that material’s electrons and atomic nuclei. These excited charges then re-emit the energy as new RF waves at the same frequency.

It would seem likely that external RF waves at 1 GHz exert stronger effects on the IWC than internal RF waves alone (5–100 MHz) in this proposed integration of RF waves with MTs. As such, external RF waves should result in greater Photonic/RF Quantum Coherence and greater quantum memory storage (Figure 12b). Certainly, external RF waves can affect tubulin units of MTs [75,76], presumably through both increased endogenous RF induction in the MTs themselves and direct vibrational effects on the IWC.

The whole concept of biophotons affecting memory is a new and exciting area of biophysics and cognitive neuroscience, which could be termed *“Biophotonic Memory”*. Biophotons are an important component of cellular electromagnetic signaling because they can carry information [77,78,79] and are the fastest carriers of quantum information [80]. Biophotons generated by MTs (specifically in their IWC) are “superluminal” and appear to be involved in quantum computations [74]. Their orbital angular momentum (twisting) relative to their direction of propagation, itself, carries information because biophotons have an infinite number of available states [80]. As mentioned earlier, photons originating from the IWC of MTs can affect tubulin qubits by exciting them to become SPQs, which then produce quantum computing and quantum memory. In this context, it has been proposed that the IWC of MTs is a “metamaterial”, with IWC-generated photons propagating to the tubulin walls of MTs to induce quantum computations for memory incorporation/storage [74].

**Figure 13:**
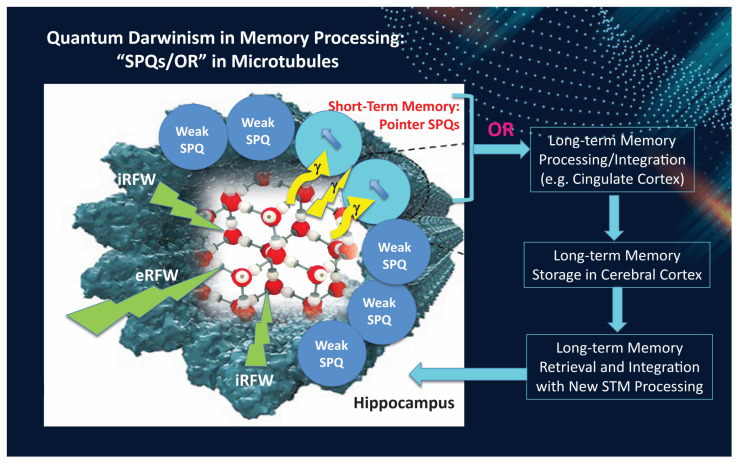
Quantum Darwinism in Memory Processing by Microtubules. A quantum memory system wherein long-term memory can only emerge if pointer SPQs (indicated by their blue arrows) can be “amplified” in short-term memory of an event that may then undergo OR to produce long-term memory. The system can be impacted by both radiofrequency waves (green) originating from tubulin wall vibrations (iRFW) and external administration (eRFW). The result would be the generation of photons and/or RF waves (yellow) within the inner water crystalline core that then interacts with tubulin to induce “most fit” pointer SPQs and “weak” SPQs. The ensuing population of pointer SPQs is proposed to result in short-term memory of an event. Generated weak SPQs of the event would dissipate quickly. Following OR of most fit pointer SPQs, long-term memory of the event is posited to integrate via neuronal pathways to the Cingulate Cortex and elsewhere, with storage in the Cerebral Cortex. Completing an anatomical feedback loop, long-term memory retrieval can be integrated with new, related short-term memory events.

Engaging Quantum Darwinism to Dissect Memory Processing. The important question now becomes: *How are superposition qubits and their objective reduction (SPQ/OR) in neuronal MTs converted into real-world (classical) short-term and long-term memory?* “Quantum Darwinism”, as proposed by Wojciech Zurek [81], may offer significant insight into this question. Quantum Darwinism is based on Darwinian natural selection, which is induced by the environment interacting with a quantum system. According to Quantum Darwinism, there are multiple possible quantum states for encoding a given (memory) event, with most of these being selected against in favor of more fit, more stable pointer states (einselection). Accordingly, a quantum system for memory must have robust SPQs in the face of disruptive environmental decoherence provided by the sea of biophotons within the IWC—a sea in which all quantum-based memory processing goes through. As such, the environmentally most fit and robust SPQs are “pointer SPQs” (Figure 13). Zurek [81] postulates that classical states (e.g., memory) are only possible because of pointer states (e.g., pointer SPQs).

It would appear that a classical world of long-term memory can only emerge from this quantum Darwinian system if pointer SPQs are strong enough to form short-term memory of an event that can then undergo OR to produce long-term memory of the event (Figure 8a,b). New pointer SPQs in MT walls are created constantly for the continuous flow of memory events through input from their environment (IWC’s photons, RF waves). Should a short-term memory event be destined for long-term memory storage, it may first be processed via neuronal pathways in integration areas (e.g., cingulate cortex) prior to final storage and later retrieval in the cerebral cortex as long-term memory (Figure 13). Through reciprocal anatomic pathways between the cerebral cortex and hippocampus, the stored memory/information could then interact later with new, related short-term memories forming in hippocampal microtubules. Such reciprocal exchange of information between the hippocampus and cerebral cortex is particularly well developed in humans. The proposed involvement of Quantum Darwinism in microtubule memory events is summarized in Figure 13. It is noteworthy that this memory processing occurs extremely rapidly, quickly making weak SPQs for any given memory event undetectable almost instantaneously.

Interestingly, photons (such as those generated in the IWC of MTs) have been counted over the heads of humans while they rest or engage in perception tasks. In Casey [82], ultraweak photon emissions (UPEs) were detected in both resting and active human brains, while perceptive tasks modified these UPEs. Indeed, a correlation exists between neuronal metabolic activity and memory/ cognition [83,84,85]. In view of externally applied RF waves at 1 GHz being able to increase EEG alpha wave (8–13 Hz) activity in humans [72,73], it should not be surprising that UPEs even have a significant phase and frequency relationship with alpha waves in humans [86]. Alpha wave activity is linked to memory and information processing, so alpha waves are highly desirable to attain memory/cognition enhancement in neurofeedback training [87]. As well, EEG alpha waves are uniquely elevated in humans during prayer, reflecting a state of calm and focused attention. For example, studies in both Catholic sisters and Muslims show this distinctive increase in alpha wave activity during prayer [88,89]. Since 1 GHz RF waves increase alpha wave activity in humans [72,73], this increase may add to the already present alpha state during prayer to accentuate the relationship between alpha waves and UPEs.

If it can be determined more precisely how RF waves, biophotons in the IWC, and MT tubulins interact through SPQ/OR, significant insight into the basis of quantum memory may be attained. Also, how SPQ/OR interacts with higher-level neuronal networks in the hippocampus and cerebral cortex to provide quantum memory is a critical subject for future empirical investigations. If the basis of memory is indeed quantum in nature as currently proposed (as opposed to “classical” neuronal communications and networks), a much faster core system of memory consolidation and retrieval would stand to be the obvious result. Along that line, a recent computer-modeled study has shown that the storage and retrieval of isometry channels (maps that encode a smaller quantum system into a larger one via quantum memory) significantly outperforms classical storage approaches [90].

It should be mentioned that others have previously proposed memory models based on quantum mechanisms. Ricciardi and Umezawa [91] first suggested use of quantum fields to describe memory. Their basic idea was to think of memory storage and retrieval in terms of non-locality and a breakdown of symmetry—in other words, they believed that memory is a diffuse activity occurring throughout the brain and, as such, that the brain has *long-range correlations* that may be induced by spontaneous breakdowns of symmetry. They describe the brain as a “macroscopic quantum system” above the level of individual cells. This theory has gone through several refinements, including a dissipative quantum model of the brain wherein memory states are constructed through condensation in the lowest energy state of quanta associated with the aforementioned long-range correlations [92,93]. These quantum theories/models of diffuse memory are theoretical in nature and are not supported by any empirical studies to our knowledge. By contrast, our SPQ/OR theory involving memory is based at the sub-cellular level within neuronal MTs through a specific group of brain areas connected to one another synaptically. Thus, SPQ/OR is quite different from and not compatible with the aforementioned dissipative quantum field models that are theoretical/mathematically-based. Moreover, in this section and the following Section 7 and Section 8 of this paper, we provide empirical evidence in the form of both pre-clinical and clinical studies that are consistent with and support the SPQ/OR theory.

In reference to the present SPQ/OR Theory of memory, a metaphor may best describe our present understanding of how RF waves, biophotons, and MT tubulins interact to result in quantum memory/cognition: *“We may know who the actors are in the play entitled ‘Human Memory and Cognition’, but not yet the actors’ scripts”*.

*Section 6 Summary*

- Both computer simulation and cell culture studies show that RF waves can provide biophysical/physiologic effects, particularly at 1 GHz frequency.
- Internal and externally applied RF waves are proposed to enter a MT’s inner water crystalline core to generate photons and/or RF waves. They, in turn, interact with the MT’s tubulins to induce SPQ formation, which provides for *“Photonic/RF wave Quantum Computation”*.
- In this proposed quantum computation, externally applied RF waves would exert stronger effects on a MT’s inner water core than internal RF waves alone.
- The principal tenets of Quantum Darwinism provide insight into how quantum interactions within MTs may transition to classical (real-world) memory. Long-term memory of an event can only emerge if “pointer” SPQs can be amplified or are strong enough for short-term memory of the event that the event may then undergo OR to produce long-term memory. This is the essence of the SPQ/OR Theory of memory.

## 7. Cognitive Benefits of Transcranial RF Wave Treatment: Three Mechanisms of Action Involving MTs

Computer modeling [70,71,94] and cell cultures [34] have provided valuable information consistent with endogenous RF waves interacting with neuronal MTs. However, *what has been lacking is clear memory-based support for this interaction in either animal studies or human clinical studies.* This section provides: (1) direct animal studies demonstrating external RF waves at 1 GHz benefit memory/cognition, (2) the epidemiologic human studies around 1 GHz that offer indirect support for memory enhancement, and (3) three mechanisms of external RF action that likely contribute to memory enhancement in normal subjects. Together, these studies reinforce this paper’s central premise that exogenous RF waves (specifically around 1 GHz) can beneficially affect the “endogenous MT/RF wave fabric” to enhance normal cognition. In so doing, these studies are consistent with SPQ/OR as the fundamental basis of memory/cognition.

### 7.1. Animal RF Wave Treatment in Cognitive Studies

**Figure 14:**
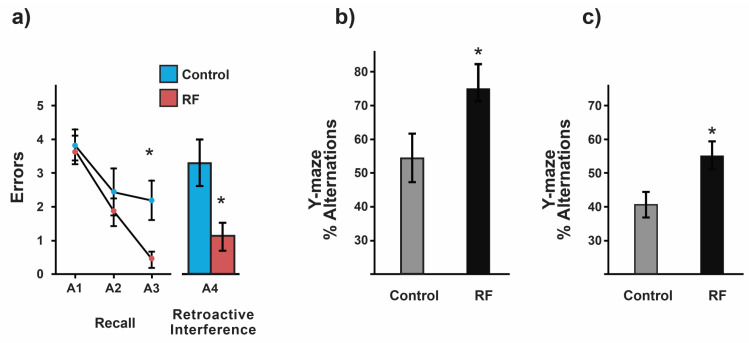
(**a**) In the difficult cognitive interference task, adult mice showed significant improvement in both the 3-trial recall and retroactive interference measures at 5 months into 1 GHz RF wave treatment. (**b**,**c**,) RF wave treatment improved Y-maze memory of adult mice at 7 months into 1-GHz RF treatment (**b**) and of aged mice at two months into 1 GHz RF treatment (**c**). Asterisks indicate *p* < 0.05 or higher level of significance vs. Controls. (Adapted from refs. [99,100]).

Most of the early work involving around 1 GHz radiofrequency treatment (0.9–1.0 GHz) to rodents reported no effects on memory/cognition [95,96,97,98]. However, such studies usually involved either acute treatment (14 days or less), low RF power levels (<0.1 W/kg), or cognitive testing conducted well after RF treatment had been completed. By contrast, our normal mouse studies described below involved cognitive testing at 5–7 months into daily RF or sham treatment, power levels around 1.0 W/kg, and cognitive testing performed between the two daily 1 h treatments. In our studies, normal adult or normal aged mice received RF treatment (near-field) via a single RF antenna placed in front of their cages that were arranged in a circle around the antenna/emitter. Mice showed cognitive improvement in both the challenging cognitive interference task of memory and the Y-maze alternation task of mnemonic memory [99,100]. Specifically, at 5 months into daily RF wave treatment, normal adult mice being tested in the cognitive interference task had much better memory performance in both 3-trial recall and retroactive interference measures (Figure 14a). In one of several separate studies evaluating memory in the Y-maze alternation task, adult mice showed significant improvement at 7 months into RF treatment (Figure 14b) [99]. Moreover, in another study involving very old normal mice, they also showed Y-maze memory enhancement after two months of daily treatment (Figure 14c) [100]. Given the above memory/cognitive benefits of long-term RF treatment in mice, the question arises as to the safety of such treatment in mice. As comprehensively reviewed by Arendash [101,102], animal studies have concluded time and time again that long-term exposure to radiofrequency waves at and around 1 GHz has no negative impact on cognition or health. This conclusion is underscored by safety endpoints evaluated in our own long-term mouse studies [99,100,101,102] wherein no effects on body/brain temperature, DNA repair, protein oxidation, antioxidants, or Alu-DNA (a marker for radiation-induced cell damage) were found. Indeed, essentially all reported deleterious effects of RF treatment in animals or cell cultures involved induction of tissue hyperthermia and/or involved only acute treatment [103,104,105].

In sharp contrast to our mouse studies utilizing 1 GHz radiofrequency to successfully enhance memory/cognition, rodent studies administering radiofrequencies of 2 GHz or higher have reported no effect or impaired cognitive function [106,107,108]. Similarly, frequencies of 50–60 Hz (via magnetic stimulation) also have no effect or impair cognition [109,110]. Such studies involving higher or lower frequencies relative to 1 GHz emphasize the requirement for use of frequencies at or around 1 GHz to achieve cognitive benefits in rodents.

### 7.2. Mechanisms for Mouse Cognitive Benefits of RF Treatment: Their Linkage to MT Function

We have identified three mechanisms that likely contribute to the memory/cognitive benefits to normal mice of applying RF waves at 1 GHz—*Neuronal MTs are central to all three of these mechanisms, as supported by the following evidence:*

**Mechanism #1: *Neuronal Activation by Increasing MT Vibrations.***

**Figure 15:**
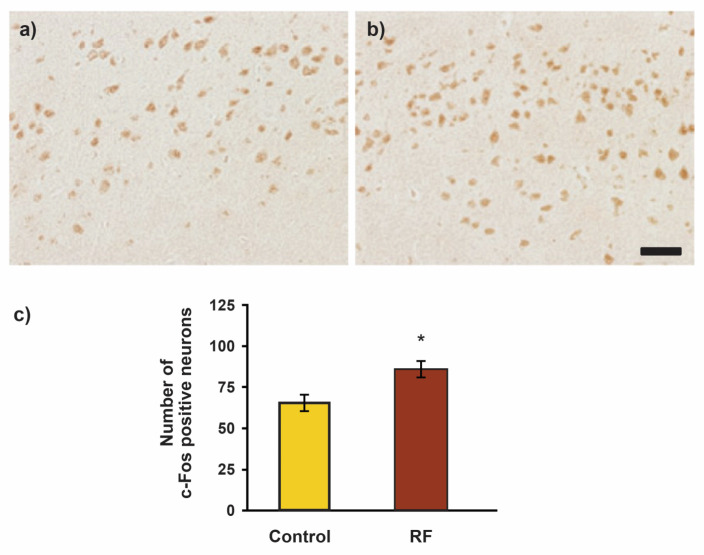
Neuronal activity (as indexed by c-Fos-positive neuronal numbers) in the entorhinal cortex of very old normal mice given two months of daily RF treatment at 1 GHz. (**a**,**b**) Photomicrographs of c-Fos-positive neurons in the entorhinal cortex from representative mice given either sham (**a**) or RF treatment (**b**). Note the greater number of c-Fos-positive neurons in the RF-treated mouse. Bar = 50 μm. (**c**) Number of c-Fos-positive neurons in the entorhinal cortex of normal mice given TRFT or sham treatment. * *p* < 0.01 (Adapted from ref. [111]).

As indicated earlier, the inner water core of MTs spontaneously vibrates at or above 1 GHz [33,40]. As such, administration of RF waves at 1 GHz would be expected to further vibrate MT inner water cores and thus entire MTs—most particularly those MTs associated with the inside of neuronal membranes through MT association proteins (MAPs; Figure 4). The enhanced vibrations provided by application of RF at 1 GHz would appear to shake these MTs from their MAPs. Since membrane-associated MTs are highly negative (hyperpolarizing), their resulting disassociation from neuronal membranes would result in less membrane hyperpolarization, more membrane depolarization, and resulting neuronal activation. Thus, it is likely that MTs can be an intermediary for converting external RF energy to neuronal activation. Evidence for this mechanism is provided by our study involving daily RF treatments at 1 GHz over a two-month period to old mice and indexing neuronal activity in their entorhinal cortex through neuronal expression of c-Fox (Figure 15). RF treatment resulted in a substantial 37% increase in neuronal activity that was linked to increased memory in the same animals [111].

**Mechanism #2: *Mitochondrial enhancement by providing energy for MT Memory Processing***

**Figure 16:**
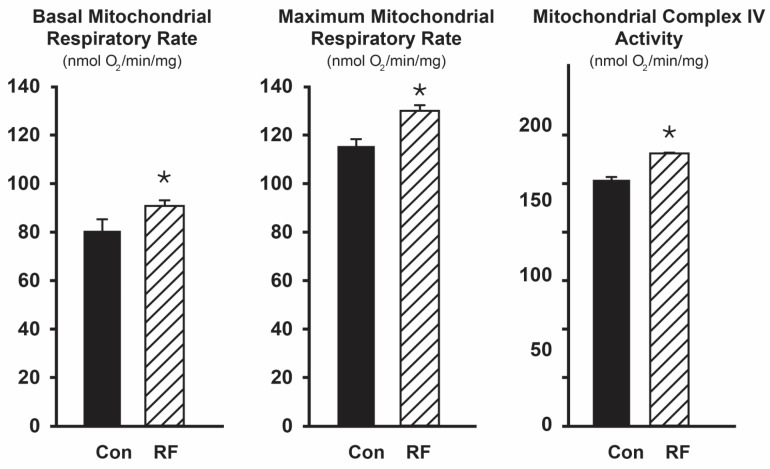
RF treatment for 1 month to aged mice significantly increased three indices of brain mitochondrial function, as measured in hippocampal mitochondria. “Con” is the control group. Asterisk indicates *p* < 0.05 or higher level of significance vs. controls. (Modified from ref. [112]).

The energy for MT vibration (and thus memory processing) comes in the form of GTP and ATP generated by adjacent mitochondria. A strong indication that mitochondrial enhancement was involved in the RF-induced cognitive benefits we observed in normal mice is provided by our study that focused on mitochondrial measures [112]. In that study, old normal mice were given daily RF treatment at 1 GHz for one month. Brain (hippocampal) analysis of *isolated neuronal mitochondria* thereafter revealed significant enhancement of mitochondrial function across three established measures—basal respiratory rate, maximum respiratory rate, and Complex IV activity (Figure 16). Although RF-induced enhancement of Complex IV activity is probably the most important measure benefited in normal mice, the exact mechanism for this Complex IV enhancement is not currently known. Certainly, these early mitochondrial benefits present at only one month of RF treatment suggest that enhancing mitochondrial function is an important early RF mechanism for providing memory benefits. In that regard, it is likely that RF-induced mitochondrial enhancement at one month and ensuing memory benefits are linked together by enhanced MT vibrational activity and memory processing.

***Mechanism #3: Induction of MT polymerization/synaptogenesis***

**Figure 17:**
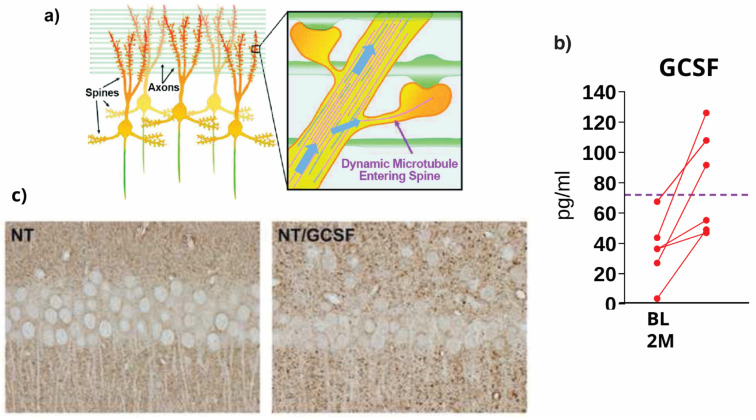
(**a**) MT polymerization resulting in synaptogenesis/synaptic remodeling with MT growth into both dendritic shafts and dendritic spines. (**b**) Plasma levels of the cytokine GCSF at baseline (BL) and at 2 months after daily transcranial RF wave treatment in AD patients. Treatment resulted in large increases in plasma GCSF levels. The dashed purple line represents normal plasma levels of GCSF. (**c**) Photomicrographic examples showing GCSF’s enhancement of synaptophysin immunostaining in the mouse hippocampus area CA1. Normal-aged (14.5monthold) mice were treated every other day with GCSF or vehicle subcutaneously for three weeks. (“(**a**)” adapted from ref. [31]; “(**b**)” and “(**c**)” adapted from refs. [119] and [120], respectively). Scale bar 50 µm.

As discussed earlier (Section 2), MTs can undergo both polymerization and depolymerization of their tubulin dimers to switch between a growing and a shrinking phase. MTs can polymerize into existing dendritic spines or form new dendritic spines to induce synaptic remodeling/synaptogenesis [113,114,115,116,117] (Figure 17a). MT stabilization and growth (polymerization) have been shown to be important for memory processing [30]. In contrast, MTs constantly or primarily in a depolymerization state can result in neurologic diseases of memory/cognition (e.g., AD) [28,31,118]. To determine the effects of RF treatment on neuritic (immature) dendrite outgrowth, human neuronal progenitor cells in culture were exposed for six days to our standard RF treatment of 1 GHz, 1 h twice daily. Thereafter, cells were quantitatively examined under a confocal microscope to evaluate neurite outgrowth. Compared to untreated neuronal cells, RF-treated cells exhibited significant increases in several measures of neurite length (in preparation). Relatedly, in an earlier study, Taghi [76] acutely exposed polymerized brain MTs to an RF field near 1 GHz and reported that RF treatment changed the structure of MTs. In addition, Sahu [40,41] applied an AC field at 3.7 MHz to tubulin molecules and showed that they began forming tubulin protofilament-like structures. Based on our findings and those of others, it is proposed that RF treatment at or near 1 GHz can benefit memory/cognition at least in part through synaptogenesis/synaptic remodeling via induction of MT polymerization.

Supporting this view are our clinical studies in AD patients given 2 months of RF treatment via Transcranial Radiofrequency Wave Treatment (TRFT; see Section 8). Large increases in blood levels of the cytokine GCSF occurred in most AD patients concurrent with reversal of their memory impairment (Figure 17b) [119]. This is important because we had shown previously that “old” normal mice given intracerebral GCSF injections experience a significant increase in synaptogenesis within their hippocampal area CA1, while also showing a reversal of their cognitive impairment (Figure 17c) [120]. Thus, TRFT appears to induce new synaptic formation through both actions on GCSF and actions on MT polymerizing/repolymerizing to induce synaptogenesis.

**Figure 18:**
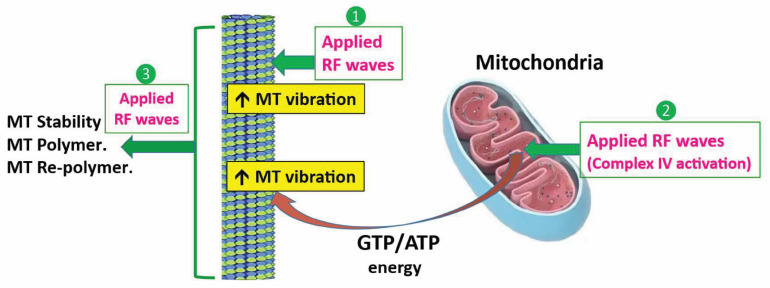
The three beneficial mechanisms of RF treatment at 1 GHz that point to neuronal MTs as cognitive enhancers. Upward arrows indicate an increase in MT vibration.

Figure 18 summarizes the multiple proposed mechanisms of RF treatment at 1 GHz that support neuronal MTs as cognitive enhancers. Specifically, RF treatment: (1) increases vibration of MTs associated with the cell membrane to likely induce increased neuronal activity, (2) increases brain mitochondrial energy production (GTP, ATP) via Complex IV activation to enhance neuronal MT vibrations, and (3) enhances dendritic growth/synaptogenesis by increasing microtubule stability, polymerization, and repolymerization. These same three mechanisms will probably also be involved in any memory/cognitive benefits observed when humans are given full-brain RF treatment at 1 GHz in future clinical studies.

### 7.3. Human Transcranial RF Wave Treatment in Cognitive Studies

Although dozens of clinical studies have investigated the potential effects of RF exposure on memory/cognition at around 1 GHz [121], these studies were always unilateral and only involved a single RF emitter on one ear via a cell phone. Moreover, the vast majority were acute studies, involving either a single exposure or several exposures within a single day. Though showing no deleterious cognitive effects, these acute studies involving very minimal RF treatment unilaterally have largely reported no effects on cognition testing conducted during or following RF exposures [102]. However, two epidemiology-based human studies have provided indirect evidence that heavy or long-term RF exposure via cell phone use in normal subjects could be beneficial to memory/cognition. First, Arns [122] found that cell phone (1 GHz) heavy users over a two-year period had better performance in a word interference test of executive function compared to moderate users or non-users. Later, Shuz [123] reported a 30–40% less risk of hospitalization for Alzheimer’s Disease or vascular dementia for cell phone (1 GHz) users of 10 years or more. Up to the present, however, no “controlled” studies in normal humans have investigated the cognitive effects of long-term, bilateral, and full-brain RF treatment over months or years. Certainly, such clinical studies at 1 GHz would have the best chance for showing memory/cognitive benefits of RF treatment—especially given the association of 1 GHz frequency with MT function. Parenthetically, any future uncontrolled normal adult studies involving 1 GHz RF treatment may provide cognitive improvement, but would not eliminate the possibility of a practice or repeat-testing effect at least partially explaining such improvement. Therefore, controlled double-blind normal human studies are required. It is also important that these clinical trials provide full-brain RF treatment.

Despite the general lack of memory/cognitive effects with acute, unilateral RF wave treatment, a number of physiologic effects have been reported in normal humans with this minimal RF treatment. First, cortical excitability is enhanced, as measured by evoked potentials [124]. Second, numerous studies have reported that acute cell phone treatment near 1 GHz enhances alpha wave activity (important for basic cognitive processing) in awake cortical EEGs [73]. Such studies suggest that neuronal activity could be beneficially enhanced by RF exposure. Since neuronal activity is coupled to glucose utilization, it is not surprising that an increase in brain glucose utilization (indexed by FDG-PET scanning) was observed in brain areas closest to a single RF emitter on the ear [125].

As was the case for rodent studies, human RF long-term treatment at 1 GHz appears to be completely safe, especially for full-brain treatments as reported in Arendash [22,23]. Moreover, all authoritative reviews have found no association of deleterious effects at or around 1 GHz with general health, subjective symptoms, cognitive function, or physiological measures [102,121,126,127]. Concerns in the early 2000s that radiofrequencies around 1 GHz could increase the risk of brain cancers from 0.5 to 1% [128,129] have been discounted by large and well-designed human epidemiologic studies performed since 2010. These latter studies have concluded time and time again that long-term exposure to RF fields at or around 1 GHz has no negative impact on the incidence of brain cancers [130,131,132,133]. In fact, Olsson et al. [134] reported that RF wave exposure at around 1 GHz is associated with significantly better survival of brain cancer (glioma) patients.

*Section 7 Summary*

- Long-term RF treatment to normal mice enhances their memory abilities, although human RF studies are yet to be carried out involving long-term, full-brain treatment.
- There are at least three mechanisms of RF wave action that likely contribute to the improved memory of mice, with MTs playing a critical role in all three of them.
- The three mechanisms of RF action are increased neuronal activation, enhanced mitochondrial function for MT energy, and increased MT polymerization/ synaptogenesis.

## 8. Treatment with Transcranial RF Waves to Stop and Reverse Alzheimer’s Memory Decline

Alzheimer’s Disease (AD) is a neurodegenerative disease characterized by a progressive loss of memory over 2–20 years due to the loss and dysfunction of neurons in brain areas important for cognition. In 2025, an estimated 7 million Americans had AD, and even more (10 million) had its prelude called mild cognitive impairment (MCI) [135]. Nearly half of people age 85 and older have the disease, while over 500,000 Americans die with AD every year. Since the early 2000s, billions of dollars have been spent in the U.S. researching traditional “pharmaceutical” therapies for AD. However, this research has been fruitless, with over 150 drugs having failed in clinical trials to prevent, stop, or reverse AD memory decline. In late 2025, for example, two major pharmaceutical companies announced clinical failure of their programs involving posdinemab (an experimental anti-tau antibody) and semaglutide (a GLP-1 drug)—both representing many millions of dollars in investment and years of work [136].

The categories of drugs currently being investigated include monoclonal antibodies against Aβ and p-tau, anti-inflammatories, senolytics, and mitochondrial enhancers. In addition, a variety of neuromodulatory approaches have been or are being tested in AD subjects, including transcranial magnetic stimulation, transcranial light/auditory stimulation, and deep brain stimulation. As with AD-tested drugs, no neuromodulatory approach has been shown to prevent, stop, or reverse the progressive memory decline of AD. Up until 2020, microtubule-targeting agents (MTAs) were also included in the AD drug category due to evidence of excess MT depolymerization in multiple neurodegenerative diseases (including AD) [28,31,118]. However, as described in Section 2, clinical trials with two MTAs in AD subjects had to be discontinued because of deleterious side effects. To stop or reverse AD progression, a therapeutic will need to target the disease process itself within neurons as described below.

### 8.1. The Primary Cause of AD: Toxic Protein Oligomers and Their Devastation of MTs

**Figure 19:**
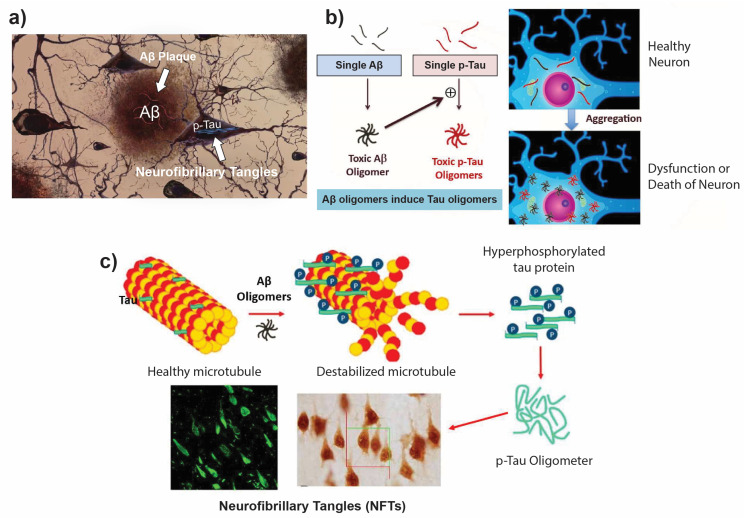
(**a**) Aggregated/insoluble Aβ and p-tau in Aβ neuritic plaques and neurofibrillary tangles, respectively, play little or no role in AD pathogenesis. (**b**) The primary culprits in AD are oligomeric Aβ and oligomeric p-tau, which form inside neurons. (**c**) The AD pathological process begins with Aβ oligomers binding to tau and phosphorylating it to p-tau, which destabilizes MTs to result in MT depolymerization and toxic p-tau oligomer formation that eventually further aggregates into insoluble/innocuous NFTs within neurons.

The aforementioned failure of drugs and neuromodulatory approaches to stop or reverse progressive AD memory loss is not due to starting therapeutic intervention too late in the disease process, as some AD researchers believe. Rather these failures are because: (1) drugs that are being clinically tested have minimal or no bioavailability within the brain’s neurons [29], and (2) essentially all AD therapeutics in clinical trials do not target the primary causes of AD, which recent studies indicate have little to do with either the large aggregates of β-amyloid (Aβ) protein outside of neurons (neuritic plaques) or intraneuronal neurofibrillary tangles (NFTs) made up of large insoluble p-tau protein deposits (Figure 19a). More specifically, monomeric Aβ is produced inside neurons, where it aggregates into Aβ oligomers to induce aggregation of monomeric p-tau into p-tau oligomers (Figure 19b). It is these two soluble, toxic “oligomers” that most recent studies conclude are the primary culprits in AD—not the insoluble Aβ plaques or NFTs [137,138,139,140,141,142] (Figure 19a).

“Intraneuronal” Aβ and tau oligomers initiate and promote AD development by causing neuronal dysfunction and death through: (1) microtubule depolymerization/destruction [143,144,145], (2) mitochondrial dysfunction resulting in less GTP/ATP [146,147] and (3) loss of dendritic spines and synapses [140,148,149]—*all three induce disruption of normal MT function related to memory/cognition.* It seems clear that any effective AD therapeutic will need to penetrate all of the brain’s neurons (AD is global) and be capable of disaggregating both Aβ and tau oligomers throughout the entire brain—targeting only Aβ oligomers may not be enough, especially after the disease has become established [150].

**Figure 20:**
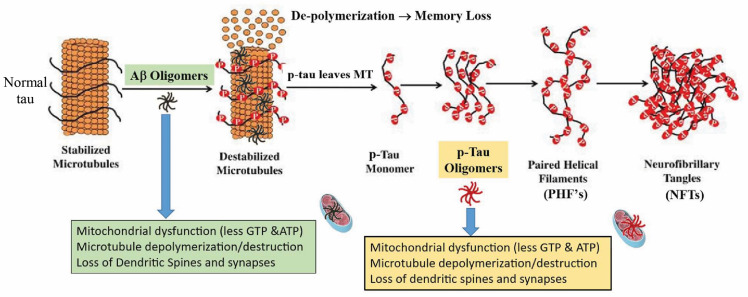
Complete AD pathologic progression within neurons, showing both p-tau-induced MT depolymerization as well as toxic effects of both Aβ and p-tau on neuronal mitochondria.

The neuropathologic process of toxic oligomer formation likely disrupts the memory function of MTs, particularly those MTs in neuronal dendrites within the cerebral cortex and hippocampus. Specifically, this process starts with Aβ oligomers targeting the normal binding of the microtubule-associated protein (MAP) “tau” to MTs (Figure 19c, upper left). These tau proteins are critical for MT stabilization and polymerization. In AD, however, toxic Aβ oligomers bind to the tau proteins of MTs and phosphorylate them to “p-tau” (Figure 19c). Because p-tau destabilizes the MT, MT depolymerization occurs, resulting in loss of MT function. Moreover, individual p-tau monomers aggregate together to form toxic p-tau oligomers that target mitochondria (see below) and eventually aggregate into insoluble/innocuous NFTs within neurons (Figure 19c, bottom). This pathologic progression is also shown in Figure 20, which not only shows the aforementioned “direct” p-tau induced MT depolymerization, but also shows the cascade of how toxic Aβ and p-tau oligomers both bind to neuronal mitochondria, resulting in: (1) mitochondrial dysfunction (less production of GTP and less ATP through the mitochondrial electron transport system) [151], (2) less GTP available for MT growth (polymerization), thus MTs shifting to depolymerization/destruction, and (3) loss of dendritic spines and synapses due to depolymerization of MTs caused by both p-tau directly and microtubule energy loss indirectly.

### 8.2. RF Treatment Studies in AD Transgenic Mice

In Section 6 and Section 7, experimental evidence was provided to support that RF waves are not only involved in MT function, but also that externally applied RF waves have beneficial effects in cell cultures and on cognition in normal rodents. However, to stop or reverse “AD” progressive memory loss, a therapeutic (or combination of therapeutics) will need to target the disease process itself within neurons—that means targeting the two toxic oligomers that cause devastation to neuronal MTs, as described in the preceding sub-section. Here, we provide the pre-clinical evidence that advanced RF wave treatment to the clinical realm of stopping and reversing the memory decline of AD, and through two well-established mouse models for AD—APPsw and APPsw + PS1 transgenic mice. These mice have had one or two human gene(s) that cause the familial (early onset) form of AD inserted into their genome at the time of egg fertilization. During adulthood, neurons within the brains of these AD transgenic mice make monomeric Aβ just as in the brains of humans with AD. Monomeric Aβ then aggregates to form toxic Aβ oligomers within neurons and further aggregates outside neurons to form (irrelevant) insoluble Aβ deposits as neuritic plaques—all as previously discussed for human AD subjects. Thus, AD transgenic mice are excellent models for AD pathogenesis related to Aβ.

**Figure 21:**
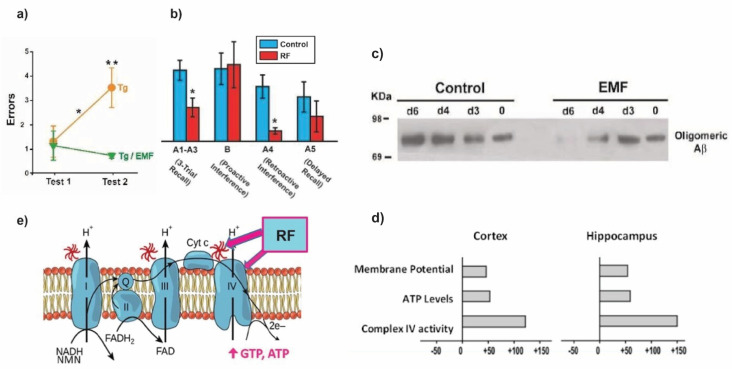
(**a**) Following baseline memory testing in young adulthood (Test 1), daily RF treatment until older age prevented the inevitable loss of memory that otherwise occurs by then (Test 2), (**b**) Old Tg mice given months of RF treatment show a reversal of their memory impairment in several measures of the cognitive interference task, (**c**) In hippocampal homogenates from Tg mice, RF treatment prevented formation of Aβ oligomers, (**d**) RF treatment for one month to aged Tg mice resulted in large increases in multiple measures of mitochondrial function in both cortex and hippocampus, (**e**) RF treatment has both a direct stimulatory effect on Complex IV activity and an Aβ-disaggregating effect on mitochondrial membranes—both increase mitochondrial energy production. EMF, RF = radiofrequency waves. Upward arrow indicates increased levels of GTP and ATP. * = *p* < 0.05 by paired t-test; ** = *p* < 0.01 for Tg vs. Tg/EMF. (**a**–**c**) adopted from ref. [99]. (**d**) adopted from ref. [112].

In an initial paper involving RF treatment to AD transgenic (Tg) mice [99], we reported that twice daily (1 h) RF treatment at 1 GHz, begun early in adulthood, protected Tg mice from otherwise certain cognitive impairment months later in a complex cognitive interference task (Figure 21a). If RF treatment to Tg mice was delayed until old age, daily RF treatment over months reversed their memory impairment in several components of the cognitive interference task (Figure 21b). In studies involving brain hippocampal homogenates from old Tg mice, a “direct” anti-aggregating effect of RF administration at 1 GHz (1 h twice daily over 6 days) prevented oligomeric Aβ formation (Figure 21c) [99]. Moreover, in aged AD transgenic mice given one month of twice-daily RF treatment at 1 GHz, large reductions in brain mitochondrial Aβ oligomers occurred, along with huge 50–150% increases in neuronal mitochondrial function—this, across multiple mitochondrial measures evaluated in both cortex and hippocampal tissue (Figure 21d) [112]. Of particular importance were direct enhancements of 120–150% in mitochondrial Complex IV Activity and 50–60% increases in ATP levels provided by RF treatment—indeed, increased mitochondrial Complex IV Activity was seen even in brains of normal mice given RF treatment as previously indicated (see Figure 16).

Considerable evidence indicates that Aβ oligomers reside on the inner mitochondrial membrane (Figure 21e), where they suppress the electron transport chain’s production of GTP and ATP, primarily by interacting with Complex IV [152,153]. Indeed, neuronal mitochondrial Aβ levels closely mirror the extent of neuronal mitochondrial dysfunction [151]. Disaggregation of these Aβ oligomers by RF treatment removes this Aβ oligomeric “brake”, resulting in profound increases in neuronal mitochondrial energy production needed for MT stability and repolymerization. The ability of RF treatment to enhance mitochondrial function can also be seen in Aβ-producing N2a cell cultures, wherein several days of RF treatment substantially increased the number of healthy mitochondrial proteins in the cell cytoplasm (unpublished results). It is apparent that an RF treatment-induced disaggregation of oligomeric Aβ on mitochondrial inner membranes [112], along with direct RF-induced Complex IV activation, results in sizable increases in mitochondrial energy production (e.g., ATP)—energy desperately needed by MTs for repolymerization and stability in the case of AD transgenic mice. It is important to underscore that the aforementioned cognitive benefits and neuronal Aβ disaggregation by RF waves in AD mice were later confirmed by four international laboratories [154,155,156,157] at similar radiofrequencies and power levels.

To summarize, RF treatment at 1 GHz to AD transgenic mice both prevents and reverses their memory/cognitive impairment, disaggregates toxic Aβ oligomers in their brains, and reverses their mitochondrial dysfunction by enhancing mitochondrial energy production needed for MT stability and growth. Therefore, the question becomes “Can these encouraging pre-clinical studies in mice be translated to human Alzheimer’s patients?” In *The Emperor’s New Mind* [43], Sir Roger Penrose first proposed a quantum involvement in human consciousness and memory. This was further expanded by his later work with Stuart Hameroff, which resulted in Orchestrated Objective Reduction [13]. The present paper further expands Orch OR into *Photonic/RF Wave Quantum Computation* as the mechanism for memory (Section 6). According to quantum computation’s dependence on vibrational/RF waves, externally applied RF waves at 1 GHz should be able to stop and possibly reverse the memory/cognitive decline of AD in humans—that is, if a device were available to provide full human brain RF treatment at 1 GHz. Fortunately, such a device has been created and utilized clinically in AD patients.

### 8.3. A Device to Administer Full-Brain RF Treatment to Humans

**Figure 22:**
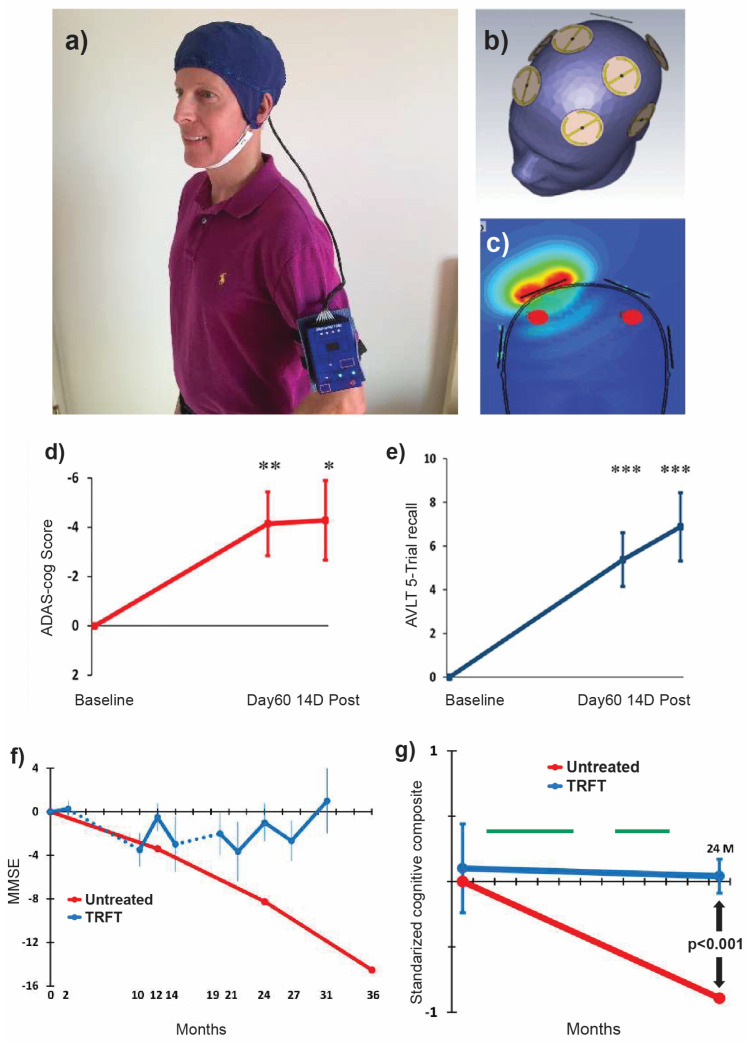
(**a**,**b**) The MemorEM (TRFT) device, as worn by a subject, has 8 RF wave emitters embedded within a 2-layered head cap, (**c**) A computer simulation shows a single emitter’s deep electric field penetration into the human brain when on, (**d**,**e**) Daily TRFT to AD subjects for several months resulted in a reversal of their cognitive impairment, as shown by both ADAS-cog and Rey AVLT testing, (**f**) During a 2½-year period of TRFT, AD subjects showed no significant decline in periodic testing in eight measures within six cognitive tasks, as exemplified by MMSE scores compared to a group of untreated AD subjects; dashed blue lines indicate the two period of no RF wave treatment, (**g**) A composite of all eight cognitive measures at 2 years into TRFT in AD subjects revealed no cognitive decline over that period, with untreated AD subjects experiencing a highly significant decline in performance of these same tasks in direct comparison to AD subjects given TRFT. * *p* < 0.05; ** *p* < 0.01; *** *p* < 0.001 compared to baseline (Adapted from refs. [22,23]).

Since our pre-clinical studies in AD transgenic mice clearly warranted clinical trials of RF wave treatment in AD subjects, G. Arendash and R. Baranowski developed and fabricated a first-of-its-kind head device (the MemorEM) to administer Transcranial Electromagnetic Wave Treatment (TEMT) in-home to human subjects [22]—the “TEMT” name has recently been changed to “TRFT” (Transcranial Radiofrequency Wave Treatment) to be more precise and is therefore utilized throughout this paper. This TRFT device is self-contained and allows for complete mobility/comfort in performing daily activities during treatment. The device has a custom printed circuit board (control panel) that is powered by a rechargeable battery. The control panel/battery box is worn on the upper arm and wired via a cable to eight specialized RF wave emitters embedded between a double-layered head cap worn by the subject (Figure 22a,b). Sequential activation at 217 Hz provides for only one emitter being on at any given time for 0.58 msec. When active, an emitter projects radiofrequency waves into the brain at 0.92 GHz (referred to as 1 GHz) and at a 1.6 W/kg power level. At this frequency and power level, human head computer simulations show that the eight emitters collectively provide penetrating TRFT to the entire human forebrain (Figure 22c). By contrast, lower or higher RFs compared to 1 GHz either go right through the human brain with minimal impact or fail to penetrate the human cranium/brain beyond the cerebral cortex, respectively. For humans, it would then appear that there is a small physiologically- and cognitively-relevant range of radiofrequencies between around 0.6 to 2.0 GHz.

We have administered TRFT at 1 GHz to human subjects for up to 2½ years without any significant side effects being reported by patients or clinicians [22,23]. Since essentially the only mechanism for any deleterious effects of RF waves is heat generation, we have put the TRFT device on a human head phantom filled with brain-analogous gel [158]. Temperatures taken throughout the brain-analogous gel before and immediately following one hour of TRFT at 4W/kg (2.5× the power in TRFT devices) resulted in none of the dozens of brain sites having a significant rise in temperature. Thus, even 2.5× the power level of current TRFT devices does not appreciably increase brain temperature (i.e., the devices are non-thermal). It should be noted that the RF/electromagnetic waves of TRFT are much different from the “magnetic” waves generated by both Transcranial Magnetic Stimulation (tMS) and pulsed electromagnetic field treatment (PEMF). In March 2020, the FDA provided “Breakthrough Device” designation for the MemorEM device—the first medical device designated for the treatment of AD memory decline. So, *can the external RF waves provided by TRFT successfully treat AD cognitive decline?*

Clinical studies provide insight into this question below.

### 8.4. Clinical Studies with TRFT to Human AD Patients

In the first study ever to administer full-brain RF waves to humans, eight mild/moderate AD patients received twice daily 1 h treatments with TRFT in-home by their caregivers for two months, utilizing the TRFT devices described above [22]. Highly significant improvements in both ADAS cog13 and Rey AVLT recall were present after 60 days of treatment (Figure 22d,e)—both of these cognitive reversals were maintained for at least 2 weeks after completion of treatment [22]. Notably, the reversal of cognitive impairment seen in ADAS cog13 (by 4 points) was equivalent to AD patients going back 1 year to the better memory/cognition they had then. In a second study involving most of the same subjects, daily TRFT was given during periods totaling 18 months over a period of 31 months (2½ years total), with periodic assessment of eight cognitive measures in six tasks [23]. One example from these eight measures that were maintained over the 2½-year treatment period by TRFT is the Mini-Mental State Score (MMSE) (Figure 22f). For comparison, another study that followed “untreated” AD patients with very similar demographics and baseline MMSE scores showed a continual decline in MMSE scores over a 3-year period [159]. In this 2½-year RF treatment study [23], a “Cognitive Composite” score made up of the same eight measures showed a complete stoppage of cognitive decline in AD patients when tested at 2 years into TRFT compared to groups of untreated AD subjects given the same cognitive tests at baseline and 2 years later (Figure 22g). Thus, continual TRFT reverses AD cognitive impairment, and periodic TRFT over 2½ years provides stable (no decline) cognitive performance. To the best of our knowledge, *these are the first clinical studies showing any AD therapeutic capable of reversing and/or stopping progressive AD cognitive decline long-term.*

### 8.5. Mechanisms of TRFT Action to Stop Memory Decline in AD Patients: MTs Are Central

**Figure 23:**
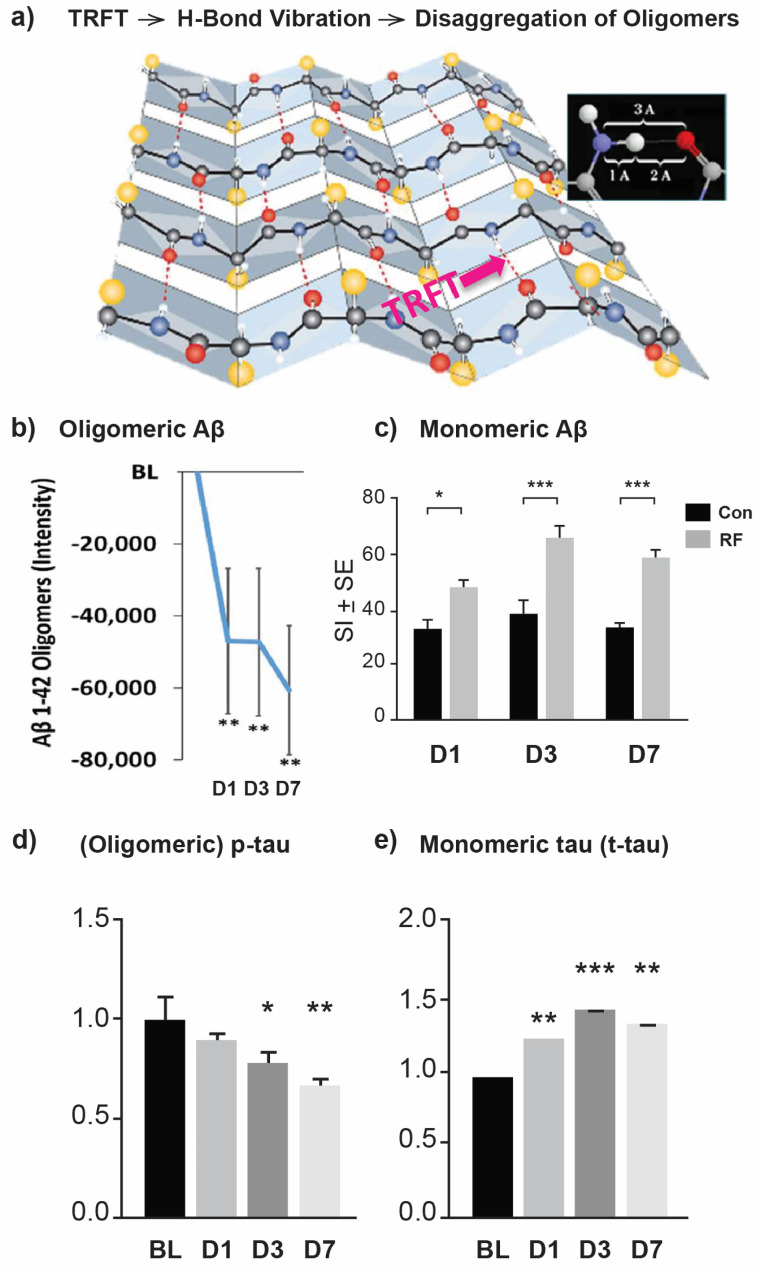
(**a**) Hydrogen bonds loosely hold individual Aβ or p-tau monomers together in forming toxic Aβ or p-tau oligomers. TRFT at 1 GHz can cause MT vibrations and reduce dipole-dipole interactions in these weak hydrogen bonds, leading to their breakage (pink arrow) and oligomer disaggregation, (**b**) In human autopsied temporal lobe homogenates, daily TRFT at 1 GHz reduced Aβ oligomer levels within 1 day, (**c**) while also increasing monomeric Aβ levels over 7 days of treatment, (**d**) In the same brain homogenates, daily TRFT at 1 Hz decreased p-tau levels (dephosphorylation) by treatment days 3 and 7 and (**e**) increased levels of monomeric tau. Thus, TRFT disaggregates both Aβ oligomers (**b**,**c**) and p-tau oligomers (**d**,**e**) in brain tissue from AD subjects. * *p* < 0.05; ** *p* < 0.01; *** *p* < 0.001 compared to controls.

As shown in Figure 20, toxic intraneuronal Aβ oligomers bind not only to mitochondria to suppress their energy production but also bind the tau proteins on MTs to phosphorylate them to “p-tau”. Because p-tau destabilizes the MT, MT depolymerization then occurs, resulting in loss of MT memory/cognitive function. Furthermore, individual p-tau monomers aggregate together forming toxic p-tau oligomers that bind to mitochondria to suppress their energy production (as do Aβ oligomers). It is important to note that both Aβ and p-tau monomers adopt a β-pleated sheet formation before they aggregate into oligomers (Figure 23a).

Oligomerization of both proteins then involves the formation of hydrogen bonds between individual Aβ or p-tau monomers. These bonds are at an abnormal angle and longer than normal protein hydrogen bonds (4.7–4.8 Å vs. 3.5–4.0 Å) [160], making them very weak and thus much more susceptible to breaking [161]. Along this line, destabilization of H-bonds between oligomer monomers occurs through dipole-dipole interactions, as well as vibration, and/or resonance phenomena [103,104,105]. RF wave frequencies around the 1 GHz frequency used in our studies have been shown to cause reduced dipole–dipole interactions (e.g., dielectric loss), which leads to a decrease in H-bonding [162,163] and consequently disaggregation of Aβ and p-tau oligomers (Figure 23a) [22,23]. Indeed, the toxic protein-sheet oligomers of Aβ and tau have a common backbone polarization that is stabilized via “two-electron interactions of H-bonds [164]—a backbone that appears to be selectively disrupted by RF waves/TRFT.

As discussed earlier in this Section, studies involving AD transgenic mice consistently show the ability of TRFT at 1 GHz to prevent Aβ oligomeric aggregation and to disaggregate already formed Aβ oligomers in their brains [99,112]. Our more recent studies (see Appendix A for methods) used autopsied human AD brain homogenates of temporal cortex to demonstrate TRFT’s ability not only to disaggregate oligomeric Aβ but also to disaggregate oligomeric p-tau [23]. For example, daily TRFT induces large decreases in oligomeric Aβ with frequencies at and around 1 GHz (Figure 23b); these decreases were near or at their maximum after only one day of TRFT. Indirectly indicative of this disaggregating action of daily TRFT are the large and highly significant increases in “monomeric” Aβ present in AD brain homogenates from 1 through 7 days into treatment at 1 GHz (Figure 23c). Mirroring these effects on Aβ disaggregation, daily TRFT induced increases in total (monomeric) tau, while also causing a “dephosphorylation” of p-tau, as indicated by a decrease in p-tau levels by Days 3 and 7 into TRFT (Figure 23d,e) [23].

**Figure 24:**
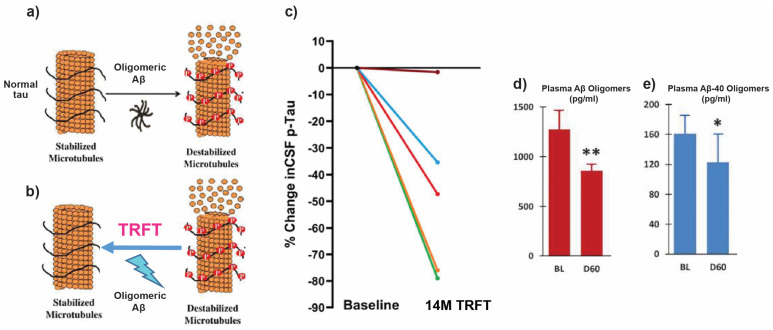
(**a**) Oligomeric Aβ induces ever-increasing levels of p-tau on MTs, leading to their destabilization/dephosphorylation. (**b**) By suppressing oligomeric Aβ levels, TRFT reverses this process to restabilize MTs. (**c**) In most AD patients, huge decreases in brain (CSF) levels of p-tau were present vs. baseline at 14 months into TRFT. Since p-tau levels in CSF are continually increasing in AD subjects, even the small decrease seen in one AD subject after 14 months of TRFT is noteworthy. (**d**,**e**) At two months into daily TRFT, plasma levels of both Aβ oligomers and soluble Aβ1-40 were significantly reduced. * Effect size greater than 0.5; ** Effect size greater 0.8 vs. baseline. (**c**) is modified from ref [23]; both (**d**) and (**e**) are modified from ref. [22].

Consistent with the aforementioned ability of TRFT to dissociate p-tau in AD brain homogenates are actions of 1 GHz TRFT in living AD subjects. As shown firstly in diagrammatic form (Figure 24a,b), brain p-tau levels continually increase in AD subjects as more and more tau on MTs is phosphorylated to p-tau by oligomeric Aβ, thus inducing MT destabilization and depolymerization (Figure 24a). However, TRFT reverses this process by decreasing oligomeric Aβ, resulting in decreased p-tau on MTs and increased monomeric t-tau for repolymerization (Figure 24b). Consistent with a TRFT-induced decrease in p-tau on MTs are the 35–79% decreases in brain (CSF) p-tau seen in most AD patients after a 14-month period of TRFT (Figure 24c) [23].

In another study [22], TRFT at 1 GHz significantly reduced Aβ oligomer and soluble Aβ levels in plasma after two months of daily TRFT (Figure 24d,e). Summarizing the above, TRFT accomplishes something that no other AD therapeutic drug or neuromodulatory device has been able to do—TRFT clinically affects all five of the major AD markers in the brain or blood of AD subjects (p-tau, t-tau, soluble Aβ1-40, soluble Aβ1-42, and oligomeric Aβ) to indeed be “disease-modifying”.

In addition to TRFT targeting the “toxic” brain oligomers of Aβ and p-tau, it could be argued that other “functionally important” oligomers may be disaggregated as well. However, functional protein oligomers have stable, compact, highly symmetrical structures with a fixed number of subunits (e.g., hemoglobin) and extensive inter-subunit contacts [103,162]. In sharp contrast, toxic oligomers (e.g., Aβ, tau) typically have a high degree of disorder and variable stoichiometry [164]—thus, TRFT is likely targeting the more disorganized “toxic” oligomers of Aβ and tau.

**Figure 25:**
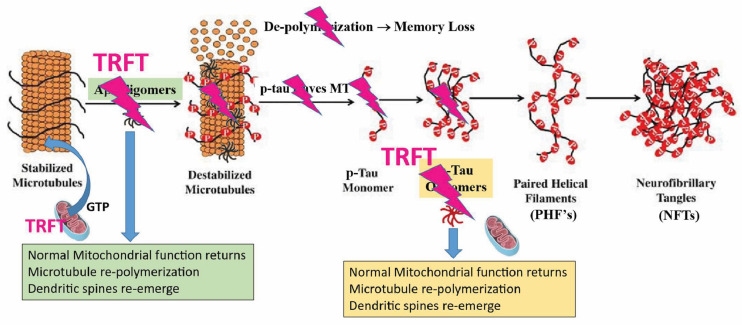
A summary of TRFT mechanisms that are likely responsible for TRFT stopping and reversing the progressive memory decline in Alzheimer’s patients. Pink lightning bolts indicate actions of TRFT.

Figure 25 summarizes the “disease-modifying” mechanisms of TRFT action that appear responsible for stopping and reversing memory/cognitive decline in Alzheimer’s patients. Firstly, TRFT disaggregates Aβ oligomers on neuronal MTs, resulting in dephosphorylation of p-tau oligomers on MTs. The result is a reversal of MT from depolymerization to MT repolymerization and stability. Secondly, TRFT disaggregates both Aβ and p-tau oligomers in neuronal mitochondria to relieve their brakes on the electron transport system (notably Complex IV). The result is increased energy (GTP, ATP) being provided to MTs for their function/vibration. The overall impact of TRFT in neurons important for memory in AD patients is likely a return of MT stability/polymerization and re-emergence of dendritic spines/synapses.

Relatedly, TRFT has several other actions that could contribute to its beneficial effects on memory/cognition in AD patients. Specifically, TRFT increases drainage of toxins/metabolites from the human brain [165,166] and rebalances the immune system’s cytokines to result in decreased brain inflammation [119]. All of these TRFT benefits are likely involved in bringing about the stoppage and reversal of AD cognitive decline.

### 8.6. FDG-PET and fMRI Benefits of TRFT in AD Patients: Linkage to Neuronal Mitochondrial and Microtubule Enhancement

**Figure 26:**
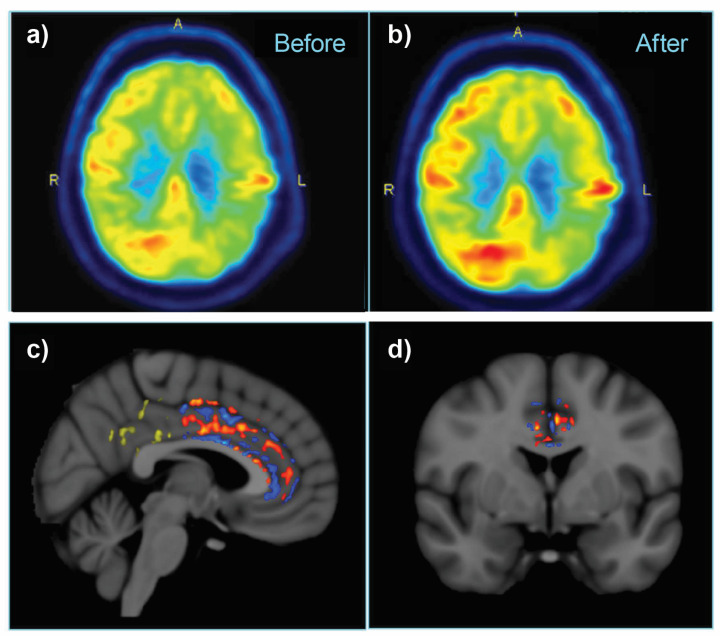
(**a**,**b**) An AD patient’s FDG-PET scan before and following two months of daily TRFT. Brain regions of greater glucose utilization/energy production, as indicated by red and yellow, are evident after TRFT. Sagittal (**c**) and frontal section (**d**) fMRI images of an AD patient’s brain showing pre- vs. post-TRFT differences in the cingulate cortex. Voxel-by-voxel analysis for significant differences (*p* < 0.05) revealed not only the expected areas of decreased fMRI in individual subjects (blue pixels), but also prominent areas of enhanced fMRI (red, orange, and yellow pixels), indicating increased neuronal communication. In untreated AD patients, such enhanced fMRI is never seen when comparing two fMRI scans separated by 2–3 months. Images taken from ref. [22].

FDG-PET brain scans show decreased glucose utilization/brain energy production at least 10 years before AD diagnosis [167,168]. Additionally, AD subjects continue to have progressively decreasing FDG-PET scanning, particularly in brain areas important for cognition (e.g., cortex, hippocampus) [169,170,171]. Moreover, AD subjects show a correlation between decreased brain FDG-PET and cognitive impairment [172]. In our own study of brain FDG-PET following daily TRFT for 2 months, we reported that some treated AD subjects showed enhanced glucose utilization (Figure 26a,b) [22], consistent with brain mitochondrial enhancement, as was seen in RF-treated AD transgenic mice [112]. For 100% of AD patients in this study, a strong correlation existed between TRFT-induced increases in brain FDG-PET and increased memory/cognitive performance following TRFT [22], indicating that the greater TRFT’s effects were on glucose utilization, the greater the memory/cognitive benefit. A second valuable brain imaging technology that we utilized is Fractional Anisotropy (FA). FA is a measure of “functional” brain MRI widely used to evaluate functional connectivity (communication) between neurons in the brain. Multiple studies have shown that brain FA (fMRI) progressively decreases in AD subjects (i.e., less neuronal communication) [173,174], especially in the cingulate cortex over a relatively short 3-month interval compared to control subjects [175]. Following 2 months of TRFT, however, 100% of AD subjects evaluated showed a dramatic enhancement in neuronal communication in their cingulate cortex (a brain area critical for memory integration) (Figure 26c,d) [22].

These increases in brain functional connectivity induced by TRFT are consistent with the aforementioned increases in brain glucose utilization in the same patients (Figure 26a,b). They are also consistent with the previously shown ability of TRFT to enhance neuronal activity (presumably by increasing MT vibrations) in mice [111]—see Section 7. Since increased neuronal activity is linked to increased glucose utilization [176], all three of these studies are strongly suggestive that a TRFT-induced increase in neuronal activity results in improved memory.

The above clinical FDG-PET and fMRI studies (and our pre-clinical work) clearly support TRFT as the first clinically effective brain “mitochondrial enhancer”—an energy (GTP, ATP) producer critically needed for MT stability and polymerization. Since brain toxic oligomer formation, ensuing mitochondrial dysfunction, and resulting energy deficits are key pathologic features in a number of neurodegenerative diseases besides AD (e.g., Parkinson’s Disease, Frontotemporal Lobe Dementia, Lewy Body Dementia, Huntington’s Disease, and ALS), therapeutic intervention with TRFT should be seriously considered beyond AD.

Interestingly, AD has been proposed to be characterized by “Brain Quantum Decoherence” due to mitochondrial and neuronal MT deterioration [177]. The authors of that paper further propose that this decoherence may be rectified by transcranial photobiomodulation (PBM) with coherent light (photons) to repair mitochondria and MTs. However, despite multiple AD clinical trials, PBM has yet to show an ability to stop or reverse memory decline in standard clinical AD tasks (i.e., MMSE, MoCA, ADAS-cog, CDR rating), nor has it been reported to affect AD markers in the brain [178,179]. By contrast, TRFT has been shown to both stop and reverse AD cognitive decline, as well as to beneficially affect levels of all major brain AD markers in small clinical studies [22,23].

**Figure 27:**
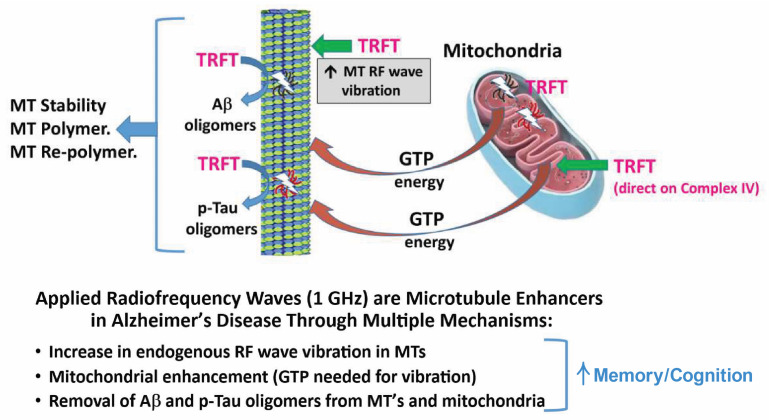
A summary showing the proposed ability of TRFT to rescue MT function in AD by removing both Aβ and p-tau oligomers from MTs and mitochondria, resulting in a return of MT stability and rescue from AD progressive memory impairment. White lightning bolts indicate actions of TRFT. Upward arrows indicate an increase in that marker.

Figure 27 is an overall summary showing: (1) the dependence of neuronal MTs on energy (GTP) provided by associated mitochondria, (2) how this relationship is deleteriously impacted in AD by Aβ and p-tau oligomers, and (3) the ability of TRFT to rescue MT function by removing both Aβ and p-tau oligomers from neuronal MTs and mitochondria. The result is likely a return of MT stability, leading to *stoppage and reversal of progressive memory/cognitive decline in AD*.

*Section 8 Summary*

- In AD, TRFT can disassociate the toxic oligomers of both Aβ and p-tau on neuronal MTs and mitochondria to increase both mitochondrial and MT function in memory-important neurons.
- TRFT can stop and reverse the progressive memory decline of Alzheimer’s Disease and could have beneficial effects on other neurodegenerative diseases that involve toxic protein oligomers.
- *TRFT appears to be a microtubule stabilizer/enhancer by decreasing p-tau in the human brain, presumably resulting in enhanced MT stabilization and MT polymerization (or repolymerization). As such, it would be the first and only clinically successful MT stabilizer in the brain.*

## 9. Limitations of This Unifying Theory of Consciousness and Memory

The presented Hybrid MT/EID theory of consciousness and SPQ/OR theory of memory collectively form a unifying theory of consciousness and memory in that MTs, their RF vibrations, and their SPQs are central to both. Although considerable detail is presented for both theories at all three anatomic levels involved (i.e., brain areas, neuronal networks, sub-cellular mechanisms), there are nonetheless limitations to this new unifying theory of consciousness and memory. Some of these limitations are indicated below:

- A primary limitation involves the clinical studies presented, in that they consisted of small numbers of AD subjects (usually 8) and did not include a control group. However, given the large number of statistically and clinically significant effects reported in these studies [22,23], it is anticipated that future clinical studies involving a larger number of subjects in a controlled design will confirm the cognitive, AD marker, and brain imaging effects of TRFT that we have reported through an “adaptive-based” single-arm design.
- There are currently only limited “quantum biophysical” data to support the unifying theories of consciousness and memory that are presented.
- Like other theories of consciousness, the currently-proposed MT/EID Theory has not yet been empirically established, but is amenable to such testing and evaluation, particularly given its defined multi-level components.
- For our SPQ/OR Theory of memory, the exact mechanism(s) of memory consolidation have yet to be defined. This includes how SPQ formation in the hippocampus/Entorhinal cortex, and Amygdala is transferred to the Prefrontal Cortex, and how the PFC engrains/holds short-term memories in preparation for their transfer and storage in the cerebral cortex.
- It has yet to be determined how the fabric of MTs, RF waves, the IWC, and SPQs (the quantum underground) interacts with higher-up neuronal networks, their electrical activity, and synapses to provide for the multiple levels of consciousness and memory.

## 10. Future Directions with TRFT as a Viable MT Enhancer in the Human Brain

This paper presents RF waves and human TRFT as the first clinically effective brain MT enhancer, and through multiple mechanisms. These important findings now provide direction to future studies that should further establish and extend our knowledge of RF waves and human TRFT as an MT enhancer—a memory enhancer that may not only improve normal human memory, but may also stop and reverse multiple neurodegenerative diseases of aging. As such, we list below five areas of future RF wave and human TRFT research:

- Neuronal cell culture studies to investigate the effects of RF waves in a range of 0.6–1.6 GHz (particularly 1 GHz) on microtubule formation and stability
- Controlled clinical trials of TRFT in normal humans to evaluate memory/cognition effects and blood measures that may reflect brain microtubule dynamics
- Controlled clinical trials of TRFT in Alzheimer’s patients to evaluate memory/cognition and brain microtubule markers. As a falsifiable prediction, we believe controlled clinical trials will show a clear, clinically significant difference in multiple tasks of memory/cognition between the TRFT and control groups.
- Further studies to test and verify the relationship between microtubules, RF waves, and photons as an interwoven fabric forming the core of human memory/cognition. As a second falsifiable prediction, we believe human administration of TRFT will increase the detection of ultraweak photon emissions (UPIs) from detectors on the scalp as evidence that TRFT-induced MT vibrations produce such emissions from the inner water core (IWC) of MTs.
- Further clinical studies in normal and AD subjects involving FDG-PET and fMRI scanning of their brains before and following TRFT during cognitive stimulation to determine which brain areas change their activity with TRFT administration. As a third falsifiable prediction, we believe brain areas that increase their activity will be those that our SPQ/OR theory indicates are involved in memory.

## 11. Summary and Conclusions

The fundamental basis of human memory and cognition has been a long-standing enigma, with various hypotheses and theories being proposed—all without much (if any) empirical evidence and most being linked to anatomic neuronal networks [2,8]. However, this broadly accepted “neuronal network” theorem cannot explain critical aspects of memory and cognition. The presented Hybrid MT/EID theory of consciousness and SPQ/OR theory of memory collectively establish a unifying theory of consciousness and memory with MTs, their RF vibrations, and SPQ formation as central to both theories. Moreover, considerable detail is provided in describing both theories at all three anatomic levels proposed (i.e., brain areas, neuronal networks, sub-cellular mechanisms). At the “intraneuronal” level, quantum processes within microtubules and their vibrations are proposed as being the basis of consciousness. Modifications to the Orch OR Theory are proposed for consciousness that incorporate the SPQ entanglement of the EID Theory without any orchestrated reduction. In then focusing on memory, the present paper provides important evidence for MTs being at the central core for memory as well. In so doing, the basic principles of Orch OR are supported, although we refined/modified Orch OR into a new basis for memory/cognition—Superposition qubits (SPQs) and Objective Reduction (OR) to account for short-term and long-term memory, respectively. Unlike other theories/hypotheses of memory, our new quantum basis for short- and long-term memory (SPQ/OR) is supported by both pre-clinical and clinical studies.

In this perspective paper, we first discussed “consciousness” in the context of forwarding a unifying quantum view of both memory and consciousness. Two quantum theories of consciousness were described—the Orch OR theory of Penrose/Hameroff [13,14,180] and the Environmental-Induced Decoherence (EID) theory of Neven and colleagues [48]. Although Superposition Qubits (SPQs) are central to both theories, the Orch OR theory is also based on the importance of MT vibrations in formation of SPQs. The EID theory postulates that Orch OR’s objective reduction (wave collapse) is not necessary for consciousness and proposes that consciousness is made continuous by the continuous quantum entanglement of constantly-forming superposition qubits/tubulins. A Hybrid (MT/EID) theory of consciousness was then proposed that proposes the Orch OR Theory’s MT vibrations to induce SPQ formation with the continual SPQ formation and SPQ entanglement of the EID Theory—all primarily within cortical Layer V pyramidal neurons. This Hybrid quantum theory of consciousness fits well into our unifying view of both memory and consciousness, as presented in this paper.

Turning then to memory at the basic level, we first proposed it to be an intraneuronal “fabric” consisting of MT tubulin walls, their vibrationally generated RF waves, and MT crystalline water cores. Primarily in cortical Layer II and III pyramidal neurons, both internal MT vibrations and externally applied RF waves would enter the MT inner water core to generate photons and/or RF waves, which in turn would interact with the MT’s tubulin units to provide for *Photonic/RF wave quantum computation*—the result is memory encoding and storage in MT tubulin units. It is this “intraneuronal fabric” that therapeutic interventions for memory enhancement or memory recovery will likely need to modulate or improve upon for their success. In this context, both computer simulation and cell culture studies show that externally applied RF waves can provide beneficial biophysic/physiologic effects, particularly at 1 GHz frequency [34,70,71]. Moreover, we proposed administration of external RF waves (particularly within the 10 MHz–1 GHz vibrational frequency of MTs) to enhance MT function or correct MT dysfunction

As inspired by Quantum Darwinism, described next was the new concept of “SPQ/OR”, involving “pointer” superposition qubits in MTs as key to short-term memory and their objective reduction for long(er) term memory storage in MTs. Consistent with this SPQ/OR theory, pre-clinical evidence was presented from cell cultures, normal mice, and AD transgenic mice demonstrating the ability of RF treatment at 1 GHz to disaggregate toxic Aβ and p-tau oligomers in the brain, enhance brain mitochondrial function, and significantly improve memory. This occurred most notably in AD transgenic mice, wherein RF treatment both protected against and treated their memory impairment. As well, evidence for three mechanisms of RF action was provided. Small clinical studies were then presented wherein AD patients were treated with TRFT, a safe and convenient biotechnology, to effectively treat the entire human brain with RF waves at 1 GHz. Long-term TRFT stopped and reversed the progressive memory decline of AD patients—*the first and only AD therapeutic to have done so through the present time.*

In the present paper, both pre-clinical and clinical studies demonstrated RF treatment’s ability at 1 GHz to address the MT and mitochondrial dysfunction in AD by directly disaggregating both Aβ and p-tau oligomers on both MTs and mitochondria. Thus, TRFT was proposed as a microtubule stabilizer/enhancer for the brain, whose actions result in enhanced MT stabilization and MT polymerization (or repolymerization). It was then proposed that 1 GHz could be a “Goldilocks” radiofrequency for benefiting the human brain in that RFs lower or higher are either not effective or risky. Parenthetically, we have evaluated many endpoints in N2a cell cultures and found that 1 GHz is better than lower frequencies (0.6 GHw) or higher frequencies (1.6 GHz) in cell survival, hippocampal function, and reduction in toxin proteins in the brain (unpublished observations). Certainly, TRFT would appear to be an ideal non-pharmacologic biotechnology to modulate the MT/RF wave vibrational fabric within neurons. Along that line, important empirical evidence has been attained for our presented SPQ/OR basis of memory. As emphasized, however, the “consciousness” component of our unifying theory (i.e., MT/EID) has not yet been empirically established, though amenable to testing and evaluation.

In summary, we have proposed that vibrational waves in MTs of cortical Layer V pyramidal cells are fundamental to human consciousness and that the resulting SPQs generated become quantum entangled with other SPQs in other MTs within the same neuron, as well as with MTs within other neurons of the brain. Moreover, we propose that memory/cognition’s deepest, intraneuronal level is a “microtubule vibrational fabric” that includes its generated vibrational/RF waves, as beneficially affected by external RF waves. In essence, this fabric is a bridge between quantum physics and the biology of life. These RF waves, particularly around 1 GHz RF, seem most important for memory processing and storage, with cortical Layer II/III neurons being critical for the latter. Indeed, the demonstrated memory/cognitive benefits provided by external RF wave administration (TRFT) may very well be based within this MT/RF wave vibrational fabric and ensuing photonic/RF wave computation. Our hybrid MT/EID hypothesis of consciousness is a reasonable combination of the best of two quantum theories, and our proposed SPQ/OR quantum theory of memory function is based on both pre-clinical studies and empirical clinical studies.

## Figures and Tables

**Figure A1:**
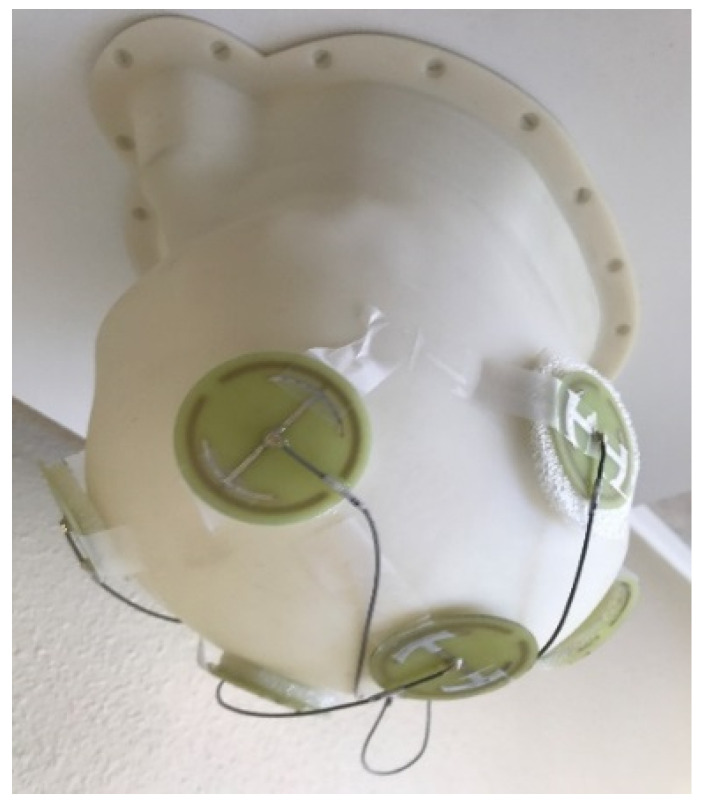
The inverted, hollow human head mannequin/phantom that was utilized in the studies of Appendix A. Shown on the outside of the head mannequin are 6 of the 8 radiofrequency wave emitters. Operating sequentially, these emitters provide RF waves at 1 GHz and at a power level of 43 V/m to the central hollow area. Eppendorf tubes containing temporal cortex homogenates from AD brains were placed within that central hollow area of the phantom from above.

## Data Availability

The new datasets used and/or analyzed during the current study are available from NeuroEM Therapeutics, Inc. upon reasonable request.

## Abbreviations

Aβ	β-amyloid
AD	Alzheimer’s Disease
EID	Environmental-Induced Decoherence
FDG-PET	Flurodeoxyglucose-positron emission tomography
fMRI	functional magnetic resonance imaging
GHz	gigahertz
IWC	inner water core
MHz	megahertz
MT	microtubule
MTAs	microtubule targeting agents
MTs	microtubules
MT/EID	Microtubule Vibrations/Environmental-Induced Decoherence (proposed MT level of consciousness)
Orch OR	Orchestrated Objective Reduction
OR	Orchestrated Reduction
PFC	Prefrontal cortex
RF	radiofrequency
SPQ	superposition qubit
SPQs	superposition qubits
SPQ/OR	superposition qubit/orchestrated reduction (proposed MT level of memory)
THz	tetrahertz
TRFT	Transcranial Radiofrequency Wave Treatment
UPEs	ultraweak photon emissions

## Appendix A

Effects of TRFT on Human Alzheimer’s Disease Autopsied Brain Tissue. The 8 antennas from a TRFT (MemorEM) head device were positioned directly on the surface of an inverted, hollow human head mannequin/phantom that is often utilized to mimic electromagnetic field exposure within the human brain (see Figure A1). Above the head phantom, Eppendorf tubes containing temporal cortex homogenates from AD brains were placed within the hollow phantom. These homogenates were then exposed to TRFT at 1 GHz and at a power level of 43 V/m (1.6 W/kg). Alternatively, a single RF antenna was utilized outside of the head device at the same frequency and power levels. TRFT was given twice daily for 1 h, and brain homogenates were analyzed in four replicates for toxic protein isoforms after 1, 3, and 7 days of treatment (the “soluble” portion of human AD brain homogenates was analyzed). These ELISA- and Western blot-based studies utilized antibodies specific for oligomeric or monomeric isoforms of Aβ, tau, and p-tau, or antibodies that bind to one of these toxic proteins within the soluble portion of AD brain homogenates. A TRFT-induced disaggregation of oligomeric isoforms would be indicated: (1) directly, by a decrease in oligomeric isoforms, or (2) indirectly, by an increase in either monomeric or soluble isoforms because a disaggregation of oligomeric/soluble isoforms into smaller/monomeric isoforms results in more antibody binding sites. Statistical analysis for treatment vs. controls for each marker involved ANOVA, with *p* < 0.05 necessary for significance.

## References

[1] Mashour G., Roelfsema P., Changeux J.-P., Dehaene S. (2020). Conscious Processing and the Global Neuronal Workspace Hypothesis. Neuron.

[2] Seth A., Bayne T. (2022). Theories of consciousness. Nat. Rev. Neurosci..

[3] Cogitate Consortium, Ferrante O., Gorska-Klimowska U., Henin S., Hirschhorn R., Khalaf A., Lepauvre A., Liu L., Richter D., Vidal Y. (2025). Adversarial testing of global neuronal workspace and integrated information theories of consciousness. Nature.

[4] Hunt T., Jones M., McFadden J., Delorme A., Hales C.G., Ericson M., Schooler J. (2024). Editorial: Electromagnetic field theories of consciousness: Opportunities and obstacles. Front. Hum. Neurosci. Sec. Cogn. Neurosci..

[5] Keppler J. (2018). The role of the brain in conscious processes: A new way of looking at the neural correlates of consciousness. Front. Psychol..

[6] Keppler J. (2025). Macroscopic quantum effects in the brain: New insights into the fundamental principle underlying conscious processes. Front. Hum. Neurosci..

[7] Verwey W. (2025). The neural basis of cognitive processing: A review and a speculative architecture. Brain Cogn..

[8] Sridhar S., Khamaj A., Astana M. (2023). Cognitive neuroscience perspective on memory: Overview and summary. Front. Hum. Neurosci..

[9] Brown R., Bligh T., Garden J. (2021). The Hebb Synapse Before Hebb: Theories of Synaptic Function in Learning and Memory Before Hebb (1949), With a Discussion of the Long-Lost Synaptic Theory of William McDougall. Front. Behav. Neurosci..

[10] Shang A., Bieszczad K. (2022). Epigenetic mechanisms regulate cue memory underlying discriminative behavior. Neurosci. Biobehav. Rev..

[11] Debanne D., Inglebert Y., Russier M. (2019). Plasticity of intrinsic neuronal excitability. Curr. Opin. Neurobiol..

[12] Zeleznikow-Johnston A., Kendziorra E., McKenzie A. (2025). What are memories made of? A survey of neuroscientists on the structural basis of long-term memory. PLoS ONE.

[13] Hameroff S., Penrose R. (2014). Consciousness in the universe: A review of the ‘Orch OR’ theory. Phys. Life Rev..

[14] Hameroff S. (2022). Consciousness, Cognition and the Neuronal Cytoskeleton—A New Paradigm Needed in Neuroscience. Front. Mol. Neurosci..

[15] Craddock T., Tuszynski J., Hameroff S. (2012). Cytoskeletal Signaling: Is Memory Encoded in Microtubule Lattices by CaMKII Phosphorylation?. PLoS Comput. Biol..

[16] Friedman G., Turk K., Budson A. (2023). The Current of Consciousness: Neural Correlates and Clinical Aspects. Curr. Neurol. Neurosci. Rep..

[17] Penrose R. (2001). Consciousness, the brain, and spacetime geometry: An addendum. Some new developments on the Orch OR model for consciousness. Rev. Ann. N. Y Acad. Sci..

[18] Penrose R., Gao S. (2022). New physics for the Orch OR consciousness proposal. Consciousness and Quantum Mechanics.

[19] Harvey R., Robinson H., Liu C., Oliva A., Fernandez-Ruiz A. (2023). Hippocampo-cortical circuits for selective memory encoding, routing, and replay. Neuron.

[20] Oliva A., Fernandez-Ruiz A., Karaba L. (2023). CA2 orchestrates hippocampal network dynamics. Hippocampus.

[21] Aru J., Suzuki M., Larkum M. (2020). Cellular Mechanisms of Conscious Processing. Trends Cogn. Sci..

[22] Arendash G., Cao C., Abulaban H., Baranowski R., Wisniewski G., Becerra L., Andel R., Lin X., Zhang X., Wittwer D. (2019). A Clinical Trial of Transcranial Electromagnetic Treatment in Alzheimer’s Disease: Cognitive Enhancement and Associated Changes in Cerebrospinal Fluid, Blood, and Brain Imaging. J. Alzheimer’s Dis..

[23] Arendash G., Abulaban H., Steen S., Andel R., Wang Y., Bai Y., Baranowski R., McGarity J., Scritsmier L., Lin X. (2022). Transcranial electromagnetic treatment stops Alzheimer’s cognitive decline over a 2½ year period: A pilot study. Medicines.

[24] Larson B., Garbus J., Pollack J., Marshall W. (2022). A unicellular walker controlled by a microtubule-based finite-state machine. Curr. Biol..

[25] Lee J., Field D., George J., Head J. (1986). Biochemical and chemical properties of tubulin subspecies. Ann. N. Y. Acad. Sci..

[26] Cyske Z., Gaffke L., Pierzynowska K., Wegrzyn G. (2023). Tubulin cytoskeleton in neurodegenerative diseases—Not only primary tubulinopathies. Cell. Mol. Neurobiol..

[27] Mitchison T., Kirschner M. (1984). Dynamic instability of microtubule growth. Nature.

[28] Boiarska Z., Passarella D. (2021). Microtubule-targeting agents and neurodegeneration. Drug Discov. Today.

[29] Brouhard G., Rice L. (2014). The contribution of ɑβ-tubulin curvature to microtubule dynamics. J. Cell Biol..

[30] Uchida S., Shumyatsky G. (2015). Deceivingly dynamic: Learning-dependent changes in stathmin and microtubules. Neurobiol. Learn. Mem..

[31] Dent E. (2017). Of microtubules and memory: Implications for microtubule dynamics in dendrites and spines. Mol. Biol. Cell.

[32] Hameroff S. (2012). How quantum brain biology can rescue conscious free will. Front. Integr. Neurosci..

[33] Pokorny J., Vrba J. (2022). Generation of Electromagnetic Field by Microtubules. Int. J. Mol. Sci..

[34] Singh P., Saxena K., Sahoo P., Ghosh S., Bandyopadhyay A. (2021). Electrophysiology using coaxial atom probe array: Live imaging reveals hidden circuits of a hippocampal neural network. J. Neurophysiol..

[35] Singh P., Manna J.S., Dey P., Sarkar S., Pattanayaka A., Nag S., Pramanik Saxena K., Krishnananda S.D., Dutta T., Bandyopadhyay A. (2024). Dodecanogram (DDG): Advancing EEG technology with a high-frequency brain activity measurement device. J. Multiscale Neurosci..

[36] Pokorny J., Jelinec F., Trkal V., Lamprecht I., Olzel R. (1997). Vibrations in microtubules. Astrophys. Space Sci..

[37] Pokorny J. (2004). Excitation of vibrations in microtubules in living cells. Bioelectrochemistry.

[38] Pizzi R., Strini G., Fiorentini S., Pappalardo V., Pregnolato M., Flores J.A. (2011). Evidence of New Biophysical Properties of Microtubules. Focus on Artificial Neural Networks.

[39] Pokorny J. (1999). Conditions for coherent vibrations in the cytoskeleton. Bioelectrochem. Bioenerg..

[40] Sahu S., Ghosh S., Ghosh B., Aswani K., Hirata K., Fujita D., Bandyopadhyay A. (2013). Atomic water channel controlling remarkable properties of a single brain microtubule: Correlating single protein to its supramolecular assembly. Biosens. Bioelectron..

[41] Sahu S., Ghosh S., Hirata K., Fujita D., Bandyopadhyay A. (2013). Multi-level memory-switching properties of a single brain microtubule. Appl. Phys. Lett..

[42] Hameroff S., Bandyopadhyay A., Lauretta D. (2026). Microtubules are ‘Fractal Time Crystals’ Implications for Life and Consciousness. J. Conscious. Stud..

[43] Penrose R., Mermin N.D. (1989). The Emperor’s New Mind: Concerning Computers, Minds, and the Laws of Physics.

[44] Hameroff S., Penrose R. (1996). Orchestrated Reduction of Quantum Coherence in Brain Microtubules: A Model for Consciousness. Math. Comput. Simul..

[45] Craddock J., Hameroff S., Tuszynski J., Poznanski R.R., Tuszynski J.A., Feinberg T.E. (2016). The ‘Quantum Underground’: Where Life and Consciousness Originate in Biophysics of Consciousness: A Foundational Approach.

[46] Hameroff S., Watt R. (1982). Information processing in microtubules. J. Theor. Biol..

[47] Craddock T.J.A., Kurian P., Preto J., Sahu K., Hameroff S.R., Klobukowski M., Tuszynski J.A. (2016). Anesthetic Alterations of Collective Terahertz Oscillations in Tubulin Correlate with Clinical Potency: Implications for Anesthetic Action and Post-Operative Cognitive Dysfunction. Sci. Rep..

[48] Neven H., Zalcman A., Read P., Kosik K.S., van der Molen T., Bouwmeester D., Bodnia E., Turin L., Koch C. (2024). Testing the Conjecture That Quantum Processes Create Conscious Experience. Entropy.

[49] Baker A., Kalmbach B., Morishima M., Kim J., Juavinett A., Li N., Dembrow N. (2018). Specialized Subpopulations of Deep-Layer Pyramidal Neurons in the Neocortex: Bridging Cellular Properties to Functional Consequences. J. Neurosci..

[50] Musall S., Sun X.R., Mohan H., An X., Gluf S., Li S.-J., Drewes R., Cravo E., Lenzi I., Yin C. (2023). Pyramidal cell types drive functionally distinct cortical activity patterns during decision-making. Nat. Neurosci..

[51] Bachmann T. (2025). Context-Sensitive Conscious Interpretation and Layer-5 Pyramidal Neurons in Multistable Perception. Brain Behav..

[52] Marvan T., Polák M., Bachmann T., Phillips W. (2021). Apical amplification—A cellular mechanism of conscious perception?. Neurosci. Conscious..

[53] Fang Z., Dang Y., Ping A., Wang C., Zhao Q., Zhao H., Li X., Zhang M. (2025). Human high-order thalamic nuclei gate conscious perception through the thalamofrontal loop. Science.

[54] Takata Y., Nakagawa H., Ninomiya T., Yamanaka H., Takada M. (2021). Morphological features of large layer V pyramidal neurons in cortical motor-related areas of macaque monkeys: Analysis of basal dendrites. Sci. Rep..

[55] Jones E. (2001). The thalamic matrix and thalamocortical synchrony. Trends Neurosci..

[56] Bachmann T., Hudetz A. (2014). It is time to combine the two main traditions in the research on the neural correlates of consciousness: C = L × D. Front. Psychol..

[57] Merker B. (2007). Consciousness without a cerebral cortex: A challenge for neuroscience and medicine. Behav. Brain Sci..

[58] Tononi G., Boly M., Massimini M., Koch C. (2016). Integrated information theory: From consciousness to its physical substrate. Nat. Rev. Neurosci..

[59] Baars B., Geld N., Kozma R. (2021). Global Workspace Theory (GWT) and Prefrontal Cortex: Recent Developments. Front. Psychol..

[60] Voss U., Holzmann R., Tuin I., Hobson J. (2009). Lucid dreaming: A state of consciousness with features of both waking and non-lucid dreaming. Sleep.

[61] Horton C. (2020). Key Concepts in Dream Research: Cognition and Consciousness Are Inherently Linked, but Do Not Control “Control”!. Front. Hum. Neurosci..

[62] LaBerge D. (2006). Apical dendrite activity in cognition and consciousness. Conscious. Cogn..

[63] Hu X., Capogna M. (2018). Dendritic inhibition in Layer 1 cortex gates associate memory. Neuron.

[64] Guan J.-S., Jiang J., Xie H., Liu K.-Y. (2016). How Does the Sparse Memory “Engram” Neurons Encode the Memory of a Spatial–Temporal Event?. Front. Neural Circuits.

[65] Spruston N. (2008). Pyramidal neurons: Dendritic structure and synaptic integration. Nat. Rev. Neurosci..

[66] Frankland P., Josselyn S., Kohler S. (2019). The neurobiological foundation of memory retrieval. Nat. NeuroSci..

[67] Guillaud L., Bosc C., Fourest-Lieuvin A., Denarier E., Pirollet F., Lafanechère L., Job D. (1998). STOP Proteins are Responsible for the High Degree of Microtubule Stabilization Observed in Neuronal Cells. J. Cell Biol..

[68] Anda F., Madabhushi R., Rei D., Meng J., Gräff J., Durak O., Meletis K., Richter M., Schwanke B., Mungenast A. (2016). Cortical neurons gradually attain a post-mitotic state. Cell Res..

[69] Damuka N., Lockhart S., Solingapuram Sai K. (2025). Imaging microtubule dynamics: A new frontier in biomarker development for neurodegenerative diseases. Alzheimer’s Dement..

[70] Havelka D., Cifra M., Kucera O. (2014). Multi-mode electro-mechanical vibrations of a microtubule: In silico demonstration of electric pulse moving along a microtubule. Appl. Phys. Lett..

[71] Havelka D., Cifra M. (2009). Calculation of the electromagnetic field around a microtubule. Acta Polytech..

[72] Vecchio F., Babiloni C., Ferreri F., Buffo P., Cibelli G., Curcio G., van Dijkman S., Melgari J.-M., Giambattistelli F., Rossini P.M. (2010). Mobile phone emission modulates inter-hemispheric functional coupling of EEG alpha rhythms in elderly compared to young subjects. Clin. Neurophysiol..

[73] Croft R., Leung S., McKenzie R., Loughran S., Iskra S., Hamblin D., Cooper N. (2010). Effects of 2G and 3G mobile phones on human alpha rhythms: Resting EEG in adolescents, young adults, and the elderly. Bioelectromagnetics.

[74] Musha T., Caligiuri L. (2015). Possible Existence of Superluminal Photons Inside Microtubules and the Resulting Explanation for Brain Mechanism. Am. J. Opt. Photonics.

[75] Pavicic I., Trosic I. (2008). In vitro testing of cellular response to ultra high frequency electromagnetic field radiation. Toxicol. Vitr..

[76] Taghi M., Gholamhosein R., Saeed R.-Z. (2013). Effect of radio frequency waves of electromagnetic field on the tubulin. Recent Pat. Endocr. Metab. Immune Drug Discov..

[77] DePaolis L., Francini R., Davoli I., De Matteis F., Scordo A., Clozza A., Grandi M., Pace E., Curceanu C., Grigolini P. (2024). Biophotons: A hard problem. Appl. Sci..

[78] Tong J. (2024). Biophoton signaling in mediation of cell-to-cell communication and radiation-induced bystander effects. Radiat. Med. Prot..

[79] Mehra J. (2021). Quantum mechanics and the explanation of life: The inclusion of human consciousness in quantum physics recognizes mind as the primary reality: Will a new science arise that can harmonize quantum physics and biology?. Am. Sci..

[80] Nevoit G., Poderiene K., Potyazhenko M., Mintser O., Jarusevicius G., Vainoras A. (2025). The concept of bioophotonic signaling in the human body and brain: Rationale, problems, and directions. Front. Syst. Neurosci..

[81] Zurek W. (2003). Decoherence, einselection, and the quantum origins of the classical. Rev. Mod. Phys..

[82] Casey H., DiBerardino I., Bonzanni M., Rouleau N., Murugan N. (2015). Exploring ultraweak photon emissions as optical markers of brain activity. iScience.

[83] Tang R., Dai J. (2014). Biophoton signal transmission and processing in the brain. J. Photochem. Photobiol..

[84] Tang R., Dai J. (2014). Spatiotemporal imaging of glutamate-induced biophotonic activities and transmission in neural circuits. PLoS ONE.

[85] Kobayashi M., Takeda M., Sato T., Yamazaki Y., Kaneko K., Ito K.-I., Kato H., Inaba H. (1999). In vivo imaging of spontaneous ultraweak photon emission from a rat’s brain correlated with cerebral energy metabolism and oxidative stress. Neurosci. Res..

[86] van Wijk R., Bosman S., Ackerman J., Van Wijk E. (2008). Correlation between fluctuations in human ultra-weak photon emission and EEG alpha rhythm. NeuroQuantology.

[87] Lavy Y., Dwolatzky T., Kaplan Z., Guez J., Todder D. (2019). Neurofeedback improves memory and peak alpha frequency in individuals with Mild Cognitive Impairment. Appl. Psychophysiol. Biofeedback.

[88] Barcelona J., Fahlman M., Churakova Y., Canjels R., Mallare J., van den Heuvel M. (2020). Frontal alpha asymmetry during prayerful and resting states: An EEG study in Catholic sisters. Int. J. Psychophysiol..

[89] Doufesh H., Ibrahim F., Ismail N., Wan Ahmad W. (2014). Effect of Muslim prayer (Salat) on α electroencephalography and its relationship with autonomic nervous system activity. J. Altern. Complement. Med..

[90] Yoshida S., Miyazaki J., Murao M. (2026). Quantum advantage in storage and retrieval of isometry channels. Phys. Rev. Lett..

[91] Ricciardi L., Umezawa H. (1967). Brain and physics of many-body problems. Kybernetik.

[92] Vitiello G. (2015). The use of many-body physics and thermodynamics to describe the dynamics of rhythmic generators in sensory cortices engaged in memory and learning. Curr. Opin. Neurobiol..

[93] Vitiello G. (2020). Matter, mind and consciousness: From information to meaning. J. Integr. Neurosci..

[94] Cifra M., Pokorny J., Havelka D., Kucera O. (2010). Electric field generated by axial longitudinal vibration modes of microtubule. BioSystems.

[95] Sienkiewicz Z., Blackwell R., Haylock R., Saunders R., Cobb B.L. (2000). Low-level exposure to pulsed 900 MHz microwave radiation does not cause deficits in the performance of a spatial learning task in mice. Bioelectromagnetics.

[96] Dubreil D., Jay T., Edeline J. (2002). Does head-only exposure to GSM-900 electromagnetic fields affect the performance of rats in spatial learning tasks?. Behav. Brain Res..

[97] Dubreuil D., Jay T., Edeline J. (2003). Head-only exposure to GSM 900-MHz electromagnetic fields does not alter rat’s memory in spatial and non-spatial tasks. Behav. Brain Res..

[98] Ammari M., Jacquet A., Lecomte A., Sakly M., Abdelmelek H., de Seze R. (2008). Effect of head-only sub-chronic and chronic exposure to 900-MHz GSM electromagnetic fields on spatial memory in rats. Brain Inj..

[99] Arendash G.W., Sanchez-Ramos J., Mori T., Mamcarz M., Lin X., Runfeldt M., Wang L., Zhang G., Sava V., Tan J. (2010). Electromagnetic field treatment protects against and reverses cognitive impairment in Alzheimer’s disease mice. J. Alzheimer’s Dis..

[100] Arendash G., Mori T., Dorsey M., Gonzalez R., Tajiri N., Borlongan C. (2012). Electromagnetic treatment to old Alzheimer’s mice reverses-amyloid deposition, modifies cerebral blood flow, and provides selected cognitive benefit. PLoS ONE.

[101] Arendash G. (2012). Transcranial electromagnetic treatment against Alzheimer’s disease: Why it has the potential to trump Alzheimer’s disease drug development. J. Alzheimer’s Dis..

[102] Arendash G. (2016). Review of the evidence that Transcranial Electromagnetic Treatment will be a safe and effective therapeutic against Alzheimer’s Disease. J. Alzheimer’s Dis..

[103] English N., Solomentsev G., O’Brien P. (2009). Non equilibrium molecular dynamics study of electric and low-frequency microwave fields on hen egg white lysozyme. J. Chem. Phys..

[104] Gerner C., Haudek V., Schandl U., Bayer E., Gundacker N., Hutter H.P., Mosgoeller W. (2010). Increased protein synthesis by cells exposed to a 1800 MHz RF mobile phone electromagnetic field, detected by proteome profiling. Int. Arch. Occup. Environ. Health.

[105] Todorova N., Bentveizen A., English N., Yarovsky I. (2016). Electromagnetic-field effects on structure and dynamics of amyloidogenic peptides. J. Chem. Phys..

[106] Wang B., Lai H. (2000). Acute exposure to pulsed 2450-MHz microwaves affects water-maze performance of rats. Bioelectromagnetics.

[107] Cosquer B., Kuster N., Cassel J. (2005). Whole-body exposure to 2.45 GHz electromagnetic fields does not alter 12-arm radial-maze with reduced access to spatial cues in rats. Behav. Brain Res..

[108] Zhao L., Peng R.Y., Wang S.M., Wang L.F., Gao Y.B., Dong J., Li X., Su Z.T. (2012). Relationship between cognition function and hippocampus structure after long-term microwave exposure. Biomed. Environ. Sci..

[109] Jadidi M., Firoozabadi S., Rashidy-Pour A., Sajadi A., Sadeghi H., Taherian A. (2007). Acute exposure to a 50 Hz magnetic field impairs consolidation of spatial memory in rats. Neurobiol. Learn. Mem..

[110] Fu Y., Wang C., Wang J., Lei Y., Ma Y. (2008). Long-term exposure to extremely low-frequency magnetic fields impairs spatial recognition memory in mice. Clin. Exp. Pharmacol. Physiol..

[111] Mori T., Arendash G. (2011). Electromagnetic field treatment enhances neuronal activity: Linkage to cognitive benefit and therapeutic implications for Alzheimer’s Disease. J. Alzheimer’s Dis. Park..

[112] Dragicevic N., Bradshaw P., Mamcartz M., Lin X., Wang L., Cao C., Arendash G. (2011). Long-term electromagnetic field treatment enhances brain mitochondrial function of both Alzheimer’s transgenic mice and normal mice: A mechanism for electromagnetic field-induced cognitive benefit?. Neuroscience.

[113] Gu J., Firestein B., Zheng J.Q. (2008). Microtubules in dendritic spine development. J. Neurosci..

[114] Hu X., Viesselmann C., Nam S., Merriam E., Dent E. (2008). Activity-dependent dynamic microtubule invasion of dendritic spines. J. Neurosci..

[115] Jaworski J., Lukas C., Kapitein S., Montenegro G., Dortland B., Wulf P., Grigoriev I. (2009). Dynamic microtubules regulate dendritic spine morphology and synaptic plasticity. Neuron.

[116] Merriam E.B., Lumbard D.C., Viesselmann C., Ballweg J., Stevenson M., Pietila L., Hu X., Dent E.W. (2011). Dynamic microtubules promote synaptic NMDA receptor-dependent spine enlargement. PLoS ONE.

[117] Hu X., Ballo L., Pietila L., Viesselmann C., Ballweg J., Lumbard D., Stevenson M., Merriam E., Dent E.W. (2011). BDNF-induced increase of PSD-95 in dendritic spines requires dynamic microtubule invasions. J. Neurosci..

[118] Sfeera A., Nicita F., Bertini E. (2020). Microtubule dysfunction: A common feature of neurodegenerative diseases. Int. J. Mol. Sci..

[119] Cao C., Abulaban H., Baranowski R., Wang Y., Bai Y., Lin X., Shen N., Zhang X., Arendash G.W. (2022). Transcranial Electromagnetic Treatment “rebalances” blood and brain cytokines levels in Alzheimer’s patients: A new mechanism for reversal of their cognitive impairment. Front. Aging Neurosci..

[120] Sanchez-Ramos J., Song S., Sava V., Catlow B., Lin X., Mori T., Cao C., Arendash G. (2009). Granulocyte colony stimulating factor decreases brain amyloid burden and reverses cognitive impairment in Alzheimer’s mice. Neuroscience.

[121] Kwon M., Hamalainen H. (2011). Effects of mobile phone electromagnetic fields: Critical evaluation of behavioral and neurophysiological studies. Bioelectromagnetics.

[122] Arns M., Van Luijtelaar G., Sumich A., Hamilton R., Gordon E. (2007). Electroencephalographic, personality, and executive function measures associated with frequent mobile phone use. Int. J. Neurosci..

[123] Schuz J., Waldemar G., Olsen J., Johansen C. (2009). Risks for central nervous system diseases among mobile phone subscribers: A Danish retrospective cohort study. PLoS ONE.

[124] Ferreri F., Curcio G., Pasqualetti P., De Gennaro L., Fini R., Rossini P. (2006). Mobile phone emissions and human brain excitability. Ann. Neurol..

[125] Volkow N.D., Tomasi D., Wang G.-J., Vaska P., Fowler J.S., Telang F., Alexoff D., Logan J., Wong C. (2020). Effects of cell phone radiofrequency signal exposure on brain glucose metabolism. JAMA.

[126] Valberg P., van Deventer T., Repacholi M. (2007). Workgroup report: Base stations and wireless networks radiofrequency (RF) exposures and health consequences. Environ. Health Perspect..

[127] Krewski D., Glickman B.W., Habash R.W.Y., Habbick B., Lotz W.G., Mandeville R., Prato F.S., Salem T., Weaver D.F. (2007). Recent advances in research on radiofrequency fields and health: 2001–2003. J. Toxicol. Environ. Health B Crit. Rev..

[128] Hardell L., Carlberg M., Sodergvist F., Hansson-Mild K. (2008). Meta-analysis of long-term mobile phone use and the association with brain tumours. Int. J. Oncol..

[129] Hardell L., Carlberg M., Hansson-Mild K. (2009). Epidemiological evidence for an association between use of wireless phones and tumor diseases. Pathophysiology.

[130] INTERPHONEStudy Group (48 collaborators) (2010). Brain tumour risk in relation to mobile telephone use: Results of the INTERPHONEinternational case-control study. Int. J. Epidemiol..

[131] Swerdlow A., Feychting M., Green A., Kheifets L., Savit D. (2011). Mobile phones, brain tumors, and the inter- phone study: Where are we now?. Environ. Health Perspect..

[132] Frei P., Poulsen A., Johansen C., Olsen J., Steding-Jessen J., Schuz J. (2011). Use of mobile phones and risk of brain tumours: Update of Danish cohort study. BMJ.

[133] Benson V., Pirie K., Schuz J., Reeves G., Berai V., Green J. (2013). Million Women Study Collaborators Mobile phone use risk of brain neoplasms other cancers: Prospective study. Int. J. Epidemiol..

[134] Olsson A., Bouaoun L., Auvinen A., Johansen C., Mathiesen T., Melin B., Lahkola A., Larjavaara S., Villegier A.-S., Byrnes G. (2019). Survival of glioma patients in relation to mobile phone use in Denmark, Finland and Sweden. J. Neurooncol..

[135] Alzheimer’s Association (USA). https://www.alz.org/alzheimers-dementia/facts-figures.

[136] Licholai G. (2025). Two Alzheimer’s Trial Failures Reveal Clinical Challenges. Forbes.

[137] Gerson J., Kayed R. (2013). Formation and propagation of tau oligomeric seeds. Front. Neurol..

[138] Pedersen J., Heegaard N. (2013). Analysis of protein aggregation in neurodegenerative disease. Anal. Chem..

[139] Goure W., Krafft G., Jerecic J., Hefti F. (2014). Targeting the proper amyloid-beta neuronal toxins: A path forward for Alzheimer’s disease immunotherapeutics. Alzheimer’s Res. Ther..

[140] Guerrero-Muñoz M., Gerson J., Castillo-Carranza D. (2015). Tau oligomers: The toxic player at synapses in Alzheimer’s Disease. Front. Cell. Neurosci..

[141] Fà M., Puzzo D., Piacentini R., Staniszewski A., Zhang H., Baltrons M.A., Puma D.D.L., Chatterjee I., Li J., Saeed F. (2016). Extracellular tau oligomers produce an immediate impairment of LTP and memory. Sci. Rep..

[142] Cline E., Bicca M., Viola K., Kirsten L. (2018). The Amyloid-β Oligomer Hypothesis: Beginning of the Third Decade. J. Alzheimer’s Dis..

[143] Zempel H., Thies E., Mankelkow E., Mandelkow E.-M. (2010). Abeta oligomers cause localized calcium elevation, missorting of endogenous tau into dendrites, tau phosphorylation, and destruction of microtubules and spines. J. Neurosci..

[144] Zempel H., Mandelkow E. (2012). Linking amyloid-beta and tau: Amyloid-beta induced synaptic dysfunction via local wreckage of the neuronal cytoskeleton. Neurodegener. Dis..

[145] Jin M., Shepardson N., Yang T., Chen G., Walsh D., Selkoe D. (2011). Soluble amyloid-beta protein dimers isolated from Alzheimer cortex directly induce tau hyperphosphorylation and neuritic degeneration. Proc. Natl. Acad. Sci. USA.

[146] Norat P., Soldozy S., Sokolowski J.D., Gorick C.M., Kumar J.S., Chae Y., Yağmurlu K., Prada F., Walker M., Levitt M.R. (2020). Mitochondrial dysfunction in neurological disorders: Exploring mitochondrial transplantation. npj Regen. Med..

[147] Bartman S., Coppotelli G., Ross J. (2024). Mitochondrial dysfunction: A key player in brain aging and diseases. Curr. Issues Mol. Biol..

[148] Lasagna-Reeves C.A., Castillo-Carranza D.L., Sengupta U., Clos A.L., Jackson G.R., Kayed R. (2011). Tau oligomers impair memory and induce synaptic and mitochondrial dysfunction in wild-type mice. Mol. Neurodegen..

[149] Umeda T., Ramser E.M., Yamashita M., Nakajima K., Mori H., Silverman M.A., Tomiyama T. (2015). Intracellular amyloid-beta oligomers impair organelle transport and induce dendritic spine loss in primary neurons. Acta Neuropathol. Comm..

[150] Mufson E., Ward S., Binder L. (2014). Prefibrillar tau oligomers in mild cognitive impairment and Alzheimer’s disease. Neurodegener. Dis..

[151] Dragicevic N., Mamcarz M., Zhu Y., Buzzeo R., Tan J., Arendash G., Bradshaw P.C. (2010). Mitochondrial amyloid-beta levels are associated with the extent of mitochondrial dysfunction in different brain regions and the degree of cognitive impairment in Alzheimer’s transgenic mice. J. Alzheimers Dis..

[152] Manczak M., Anekonda T., Henson E., Park B., Quinn J., Reddy P. (2006). Mitochondria are a direct site of A- beta accumulation in Alzheimer’s disease neurons: Implications for free radical generation and oxidative damage in disease progression. Hum. Mol. Genet..

[153] Knott A., Perkins G., Schwarzenbacher R., Bossy-Wetzel E. (2008). Mitochondrial fragmentation in neurodegeneration. Nat. Rev. Neurosci..

[154] Banaceu S., Banasr S., Sakly M., Abdelmelek H. (2013). Whole body exposure to 2.4 GHz WIFI signals: Effects on cognitive impairment in adult triple transgenic mouse models of Alzheimer’s disease (3xTg-AD). Behav. Brain Res..

[155] Jeong Y., Kang G.-Y., Kwon J., Choi H.-D., Pack J.-K., Kim N., Lee Y.-S., Lee H.-J. (2015). 1950 MHz electromagnetic fields ameliorate A-beta pathology in Alzheimer’s disease mice. Curr. Alzheimer Res..

[156] Son Y., Kim J.S., Jeong Y.J., Jeong Y.K., Kwon J.H., Choi H.-D., Pack J.-K., Kim N., Lee Y.-S., Lee H.-J. (2018). Long-term RF exposure on behavior and cerebral glucose metabolism in 5xFAD mice. Neurosci. Lett..

[157] Zhi W., Zou Y., Ma L., He S., Guo Z., Zhao X., Hu X., Wang L. (2023). 900 MHz electromagnetic field exposure relieved AD-like symptoms on APP/PS1 mice: A potential non-invasive strategy for AD treatment. Biochem. Biophys. Res. Commun..

[158] Baranowski R., Amschler J., Wittwer D., Arendash G. (2025). Memory enhancement by transcranial radiofrequency wave treatment occurs without appreciably increasing brain temperature. Phys. Eng. Sci. Med..

[159] Tan K.-S., Libonc D., Rascovsky K., Grossman M., Xie S. (2013). Differential longitudinal decline on the mini-mental state examination in Frontotemporal Lobar Degeneration and Alzheimer’s Disease. Alzheimer Dis. Assoc. Disord..

[160] Jeffrey G. (1997). An Introduction to Hydrogen Bonding.

[161] Sunde M., Serpell L., Bartlam M., Fraser P., Pepys M., Blake C. (1997). Common core structure of amyloid fibrils by synchrotron X-ray diffraction. J. Mol. Biol..

[162] Segwa R., Kaur K. (1999). Microwave absorption in oligomers of ethylene glycol. Indian J. Biochem. Biophys..

[163] Blank M., Goodman R. (2004). Initial interactions in electromagnetic field-induced biosynthesis. J. Cell. Physiol..

[164] Ciepak A. (2017). Protein folding, misfolding, and aggregation: The importance of two-electron stabilizing interactions. PLoS ONE.

[165] Arendash G., Lin X., Cao C. (2024). Enhanced brain clearance of Tau and Aβ in Alzheimer’s patients by Transcranial Radiofrequency Wave Treatment: A central role of VEGF. J. Alzheimer’s Dis..

[166] Arendash G. (2025). The Brain Toxin Cleansing of Sleep Achieved During Wakefulness. J. Clin. Med..

[167] Landau S.M., Harvey D., Madison C.M., Koeppe R.A., Reiman E.M., Foster N.L., Weiner M.W., Jagust W.J., The Alzheimer’s Disease Neuroimaging Initiative (2011). Associations between cognitive, functional, and FDG-PET measures of decline in AD and MCI. Neurobiol. Aging.

[168] Younes L., Albert M., Moghekar A., Soldan A., Pettigrew C., Miller M. (2019). Identifying Changepoints in Biomarkers during the preclinical phase of Alzheimer’s Disease. Front. Aging Neurosci..

[169] Reiman E.M., Caselli R.J., Yun L.S., Chen K., Bandy D., Minoshima S., Thibodeau S.N., Osborne D. (1996). Preclinical evidence of Alzheimer’s disease in persons homozygous for the epsilon 4 allele for apolipoprotein E. N. Engl. J. Med..

[170] Reiman E.M., Chen K., Alexander G.E., Caselli R.J., Bandy D., Osborne D., Saunders A.M., Hardy J. (2004). Functional brain abnormalities in young adults at genetic risk for late-onset Alzheimer’s dementia. Proc. Natl. Acad. Sci. USA.

[171] Chen K., Langbaum J.B., Fleisher A.S., Ayutyanont N., Reschke C., Lee W., Liu X., Bandy D., Alexander G.E., Thompson P.M. (2010). Twelve-month metabolic declines in probable Alzheimer’s disease and amnestic mild cognitive impairment assessed using an empirically pre-defined statistical region-of-interest: Findings from the Alzheimer’s Disease Neuroimaging Initiative. Neuroimage.

[172] Ferris S.H., de Leon M.J., Wolf A.P., Farkas T., Christman D.R., Reisberg B., Fowler J.S., MacGregor R., Goldman A., George A.E. (1980). Positron emission tomography in the study of aging and senile dementia. Neurobiol. Aging.

[173] Bubb E., Metzler-Baddeley C., Aggleton J. (2018). The cingulum bundle: Anatomy, function, and dysfunction. Neurosci. Biobehav. Rev..

[174] Wang T., Xiao S., Liu Y., Lin Z., Su N., Li X., Li G., Zhang M., Fang Y. (2014). The efficacy of plasma biomarkers in early diagnosis of Alzheimer’s disease. Int. J. Geriatr. Psychiatry.

[175] Nowrangi M.A., Lyketsos C.G., Leoutsakos J.S., Oishi K., Albert M., Mori S., Mielke M.M. (2013). Longitudinal, region-specific course of diffusion tensor imaging measures in mild cognitive impairment and Alzheimer’s disease. Alzheimer’s Dement..

[176] Magistretti P. (2006). Neuron-glia metabolic coupling and plasticity. J. Exp. Biol..

[177] Nichols T., Berman M., Tuszynski J. (2023). Is Alzheimer’s disease a manifestation of brain quantum decoherence resulting from mitochondrial and microtubular deterioration?. J. Multiscale Neurosci..

[178] Chan D., Suk H.-J., Jackson B.L., Milman N.P., Stark D., Klerman E.B., Kitchener E., Avalos V.S.F., de Weck G., Banerjee A. (2022). Gamma frequency sensory stimulation in mild probable Alzheimer’s dementia patients: Results of feasibility and pilot studies. PLoS ONE.

[179] Chan D., de Weck G., Jackson B.L., Suk H., Milman N.P., Kitchener E., Avalos V.S.F., Quay M.J., Aoki K., Ruiz E. (2025). Gamma sensory stimulation in mild Alzheimer’s dementia: An open-label extension study. Alzheimers Dement..

[180] Hameroff S., Penrose R. (2014). Reply to criticism of the ‘Orch OR qubit’ Orchestrated objective reduction’ is scientifically justified. Phys. Life Rev..

